# Co-Creation of Mental Health Intervention for Adolescents: A Social Hackathon Approach

**DOI:** 10.3390/healthcare14101315

**Published:** 2026-05-12

**Authors:** Hannes Baumann, Anna-Maria Ksiezarczyk

**Affiliations:** 1 Institute for Movement Therapy and Movement-Oriented Prevention and Rehabilitation, German Sport University Cologne (DSHS), 50933 Cologne, Germany; 2 Department of Sports and Exercise Medicine, Institute of Human Movement Science, University of Hamburg, 20148 Hamburg, Germany; anna-maria.ksiezarczyk@stud.uke.uni-hamburg.de

**Keywords:** student involvement, emotional integrity, mental integrity, mental soundness, codetermination, project development, exchange students, societal issues, international cohort, digital health, transdisciplinary, public health, challenge, implementation

## Abstract

**Highlights:**

**What are the main findings?**

- Adolescents in an international exchange student cohort prioritized mental health topics such as self-image, stress/anxiety, belonging, and harassment when co-creating intervention ideas. Many proposed solutions were low-threshold, social, and peer-focused, while some also incorporated movement- or sport-related elements.
- Projects that progressed further combined a clear solution concept with credible implementation detail, especially around staffing and funding.

**What are the implications of the main findings?**

- Social hackathons may be useful not only as a research tool but also as a participatory format to generate locally grounded, youth-aligned prevention ideas.
- Future intervention development should preserve adolescent-generated solution logics while strengthening implementation planning and evaluation.

**Abstract:**

**Background/Objectives**: Adolescent mental health problems emerge early, remain undertreated, and are shaped by diverse contextual stressors. In response to calls for more youth-centered prevention, school-based health promotion, and participatory intervention design, this study explored which mental health-related problems internationally mobile adolescents prioritize and which solution ideas they generate in a structured co-creation setting, including where movement- and sport-related elements are embedded. **Methods**: A qualitative, participatory study was conducted during a 24 h social hackathon embedded in the Youth Empowerment Seminar for exchange students. Hackathon materials from 43 projects were analyzed using content-structuring qualitative content analysis following Kuckartz. **Results**: Adolescents most frequently framed problems in terms of self-image, stress and anxiety, belonging, and harassment. Solutions clustered around low-threshold group formats, while implementation segments focused strongly on staffing, funding, barriers, and feasibility. Cross-domain analyses suggested recurring problem-solution matches, such as loneliness with hobby or interest groups. **Conclusions**: Social hackathons can surface adolescent-prioritized mental health concerns and translate them into context-sensitive prevention ideas. The findings mainly point to social and psychosocial solution pathways, while some proposals additionally positioned shared activity or movement contexts as potentially supportive for well-being. These results provide a starting point for subsequent school-based prototyping and feasibility work.

## 1. Introduction

Adolescence is a critical period for mental health, as many mental disorders first emerge during these years [1,2]. Globally, around one in seven adolescents aged 10–19 years experiences a mental disorder, and depression and anxiety are among the leading causes of illness and disability in this age group [1]. Large-scale meta-analytic evidence further indicates that nearly half of lifetime mental disorders begin by age 18, underscoring the importance of early prevention and timely support [2]. Untreated mental health problems in adolescence can impair physical health, educational attainment, social participation, and later occupational functioning, making adolescent mental health a major public health priority with long-term consequences [1,3].

### 1.1. Target Group and Preventive Approaches

This also highlights why interventions that directly target peer interaction and social environments, such as physical activity settings or participatory group formats, may be particularly suited to address these challenges. Adolescents are particularly vulnerable to mental health problems because this developmental stage involves major emotional, social, and identity-related transitions [1]. Social stressors such as loneliness, bullying, exclusion, and victimization can further intensify vulnerability. Recent evidence from school-aged populations shows that loneliness and bullying remain common experiences, and both are closely linked to poorer mental health outcomes [4,5,6]. Meta-analytic findings indicate that bullying is associated with substantially higher depressive symptom burden in children and adolescents, while loneliness is consistently related to poorer well-being and more internalizing symptoms [5,6]. These patterns help explain why prevention efforts increasingly focus on school and community contexts where peer relationships, belonging, and day-to-day stressors are shaped. More broadly, systematic review evidence suggests that early preventive interventions can reduce depressive and anxiety symptoms, but effects vary by delivery mode, target group, and implementation conditions [7]. Viewed through a physical activity lens, these stressors are relevant not only because movement can benefit mental health, but also because school, sport, and recreational settings are key social environments in which body image, peer comparison, belonging, and exclusion are enacted. In the conceptual model proposed by Lubans et al., such effects are expected to operate especially through psychosocial pathways, including physical self-perceptions, social connectedness, mood, and coping-related processes, while outcomes are further shaped by the type, context, and individual experience of activity [8,9,10,11,12]. Subsequent evidence increasingly supports these pathways. Review-level syntheses indicate that self-esteem, self-concept, self-efficacy, social support/social connectedness, and self-regulation are among the most consistent psychosocial mechanisms linking physical activity with psychiatric symptoms and mental well-being in young people [9,10,11,13]. In cohort work, self-esteem has been shown to mediate the association between sports participation in childhood and later internalizing symptoms, while more recent evidence suggests that using physical activity as a coping strategy may partly explain longer-term mental health benefits of team sport participation [13,14]. These mechanisms are directly relevant to the present study because many adolescent-generated ideas centered less on exercise dose and more on belonging, confidence, safe spaces, shared routines, and manageable coping opportunities. Evidence from sport settings also indicates that bullying and exclusion can occur in physical education and organized activity settings and are associated with poorer psychosocial outcomes, suggesting that prevention should explicitly address peer climate in these contexts [4,5]. Sport and physical activity can therefore represent one concrete context in which belonging, bullying, and self-esteem processes become especially visible for adolescents, making them relevant fields for preventive mental health promotion [8,15].

### 1.2. Effectiveness of Existing Approaches

Importantly, these findings underline the need for approaches that are better aligned with adolescents’ needs and real-world contexts. This perspective is particularly relevant for school-based prevention, where physical activity can be embedded in everyday routines, peer interaction, and broader health promotion structures rather than delivered as an isolated add-on. Reviews indicate that school-based physical activity programs can improve activity levels and fitness, and conceptual work on physical literacy suggests that confidence, competence, motivation, and participation may jointly support long-term well-being in children and adolescents [10,16,17,18]. The effectiveness of current adolescent mental health interventions varies, and research findings present a mixed picture. Systematic reviews and meta-analyses suggest that some school-based and targeted prevention approaches can reduce depressive and anxiety symptoms, particularly when programs are delivered to young people with elevated risk or early symptoms rather than universally to all students [7,19,20]. At the same time, average effects are often small, heterogeneity across intervention types is substantial, and many studies offer limited long-term follow-up, making it difficult to identify which approaches produce durable benefit across settings [7,19,20]. This has led to repeated calls for stronger contextual tailoring, better implementation planning, and more rigorous evaluation of sustained outcomes rather than short-term symptom change alone [7,19]. Importantly, sport or physical activity should therefore not be treated as a universal solution in itself but as one possible delivery mode whose relevance depends on the needs and preferences articulated by young people in a given context [8,15]. Long-term perspectives remain important because regular participation in physical activity has been linked to broader developmental benefits for mental health, self-esteem, and social functioning across adolescence [8,15]. Because the present study used a qualitative co-creation design, it cannot determine long-term mental health effects or pre-post changes in symptom burden; instead, it contributes insight into how adolescents themselves define relevant problems, acceptable formats, and feasible implementation conditions. Physical activity interventions have repeatedly been associated with small but meaningful improvements in self-esteem, depressive symptoms, and psychological well-being in young people, although effect sizes vary across settings and intervention types [8,15,21].

### 1.3. Digitalization and Societal Factors

The mixed engagement data further underline the need for complementary approaches that actively involve adolescents in shaping solutions that reflect their lived experiences and contextual barriers. The rapid digitalization of society in recent years has become a double-edged sword for adolescent mental health. On one side, digital technology offers new pathways for psychoeducation, screening, low-threshold support, and service access, and systematic review evidence suggests that digital mental health interventions can improve reach and offer acceptable forms of support for many adolescents and young adults [22,23,24]. However, real-world use remains challenging: dropout and non-completion are common, especially for self-guided interventions delivered outside tightly controlled study settings [25]. At the same time, broader digital environments may introduce new risks. Systematic reviews indicate that social media use can be associated with depression, anxiety, and psychological distress in adolescents, although causal pathways remain complex, and not all digital engagement is harmful [26,27]. Taken together, the literature suggests that digital formats are promising but insufficient on their own; they need careful design, sustained engagement strategies, and sensitivity to social context and stigma [22,25,26,27]. This is also reflected in mHealth research targeting lifestyle behaviors such as physical activity, sedentary behavior, sleep, and nutrition, where effects on adolescent emotional and behavioral outcomes appear promising but heterogeneous and dependent on intervention design and engagement [28]. The mixed engagement data further underline the need for complementary approaches that actively involve adolescents in shaping solutions that reflect their lived experiences and contextual barriers [22,25].

### 1.4. Critical Aspects of Current Approaches

Despite the variety of interventions available, several critical shortcomings limit the impact of current adolescent mental health approaches. First, many interventions are still developed with limited adolescent involvement, even though co-design and participatory development are increasingly recognized as important for improving relevance, acceptability, and contextual fit [23,29,30,31,32,33,34]. Second, adherence and engagement remain persistent challenges, particularly for digital interventions, where uptake in routine settings often falls well below levels observed in tightly controlled trials [22,25]. Third, implementation barriers such as limited staff capacity, competing school priorities, weak contextual fit, and insufficient planning for sustainment can undermine otherwise promising program ideas [29,35]. Together, these gaps help explain why the field continues to call for more inclusive, engaging, and implementation-aware prevention formats that move beyond top-down program design [29,30,31,35]. Co-design is gaining visibility in youth mental health research, but routine and meaningful involvement of adolescents across the full development pipeline remains uneven, especially outside specialized digital or implementation projects [30,31]. Accordingly, the present study not only asks which mental health topics adolescents prioritize but also which ideas could plausibly be translated into movement-oriented, school-based, or hybrid interventions that promote both psychological well-being and active living [8,11,12].

### 1.5. Innovation and Research Approach

Beyond existing co-design and hackathon approaches, this study focuses not only on idea generation but also on how adolescents link problems, proposed solutions and implementation considerations within a structured process. Based on students’ pre-screening responses, the authors developed a set of overarching themes that guided the hackathon. Together with structured tasks and sequential rounds, this provided a framework for the process. Within this structure, adolescents actively generated and developed intervention ideas, while researchers facilitated the process and subsequently analyzed the resulting data. In light of the above, the present work adopts an innovative approach to adolescent mental health prevention, emphasizing co-creation, agility, and user-centered design. Specifically, we use a social hackathon format to elicit youth-generated priorities, solution logics, and feasibility considerations in a time-bounded but collaborative setting [36,37]. Beyond existing co-design and hackathon approaches, this study focuses not only on idea generation but also on how adolescents link problems, proposed solutions, and implementation considerations within a structured process. Based on students’ pre-screening responses, the authors developed a set of overarching themes that guided the hackathon. Together with structured tasks and sequential rounds, this provided a framework for the process. Within this structure, adolescents actively generated and developed intervention ideas, while researchers facilitated the process and subsequently analyzed the resulting data. Our research questions therefore ask (1) which mental health-related problems adolescents consider most relevant, (2) which solutions they propose and which appear most feasible, and (3) which aspects and barriers they considered when shaping a promising project. This study responds to repeated calls for more participatory and context-sensitive intervention development pathways [29,30,31]. Rather than assuming that one intervention format will fit all adolescents, the hackathon logic acknowledges that problems and acceptable solutions may vary by school, peer culture, and local resources. In this sense, hackathons may function not only as a research method but also as a participatory mechanism for identifying context-sensitive bottom-up solutions [37,38].

## 2. Materials and Methods

### 2.1. Study Design and Reporting

This study used a qualitative, participatory design embedded in a social hackathon to elicit adolescent-generated problem framings and solution concepts for mental health promotion and resilience. A hackathon is a time-bounded, collaborative event in which participants work in small teams to develop innovative ideas or prototypes around a defined topic of interest [36,37]. Hackathons are characterized by intensive, continuous teamwork within a limited timeframe. Originating in technology and software development, hackathons have been adapted for social, health-related, and educational contexts; in these settings, social hackathons emphasize co-creation and practical, context-sensitive solutions. Health-oriented hackathon studies further show that such formats can support interdisciplinary collaboration, rapid idea development, prototype-oriented innovation, and, when accompanied by structured follow-up, translation of ideas beyond the event itself [39,40,41]. The analytic approach was qualitative content analysis with content-structuring procedures following Kuckartz, combining deductive category development with inductive refinement of subcategories through iterative coding cycles [42,43]. Reporting was guided by the Consolidated Criteria for Reporting Qualitative Research (COREQ) to ensure a complete and transparent description of the research team, study context, sampling, data sources, and analysis procedures [44]. Given the increasing attention to the quality and reporting of youth co-design processes, we treated the hackathon not only as an ideation event but also as a structured participatory research setting in which process transparency and contextual fit mattered alongside creativity [23,32,33,34].

### 2.2. Setting, Participants, Ethics and Procedure

Data were generated during the Youth Empowerment Seminar (YES), a four-day residential seminar for international high school exchange students hosted by Youth for Understanding (YFU).

**Figure 1:**
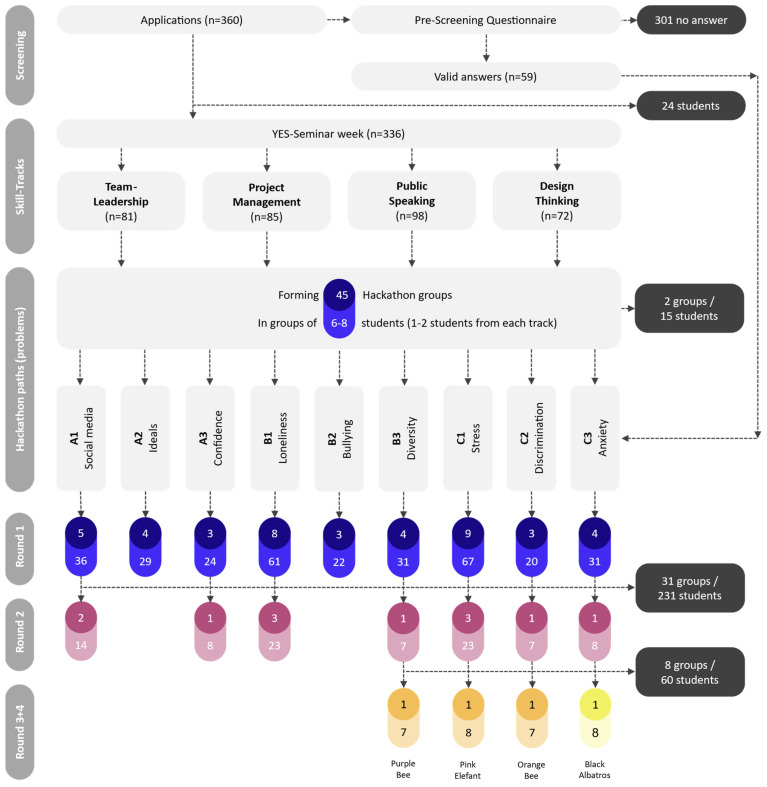
Flow of participants.

International high school students, living with host families and attending local schools, before returning to their home countries after the seminar. Three students spent about one year abroad in a host country prior to the seminar, lived with a host family, and attended a foreign high school. After the seminar, students returned to their home country. The seminar took place in June 2023 at a retreat venue at Werbellinsee (Brandenburg, Germany). Participants stayed in shared, gender-segregated rooms (up to six adolescents). Participant flow followed three denominators reflecting recruitment, attendance, and analytic inclusion. While the hackathon followed a participatory co-creation approach, the process was deliberately structured through predefined themes (based on the screening questionnaire), guided tasks, and sequential rounds to support idea development and comparability across groups. Students represented diverse countries and school systems, reflecting both home and host country contexts. During team-working time, the staff was distributed across the venue in several booths, consisting of expert booths (leaders of previously completed skill tracks) and general consultation booths (e.g., if in need of more material or general questions related to Hackathon procedure). First, all seminar applicants (*n* = 360) were invited to complete a brief pre-seminar screening questionnaire; 59 responded and 301 did not respond (for details about the questionnaire and resulting data, see Supplementary Materials S1). Second, 336 adolescents attended the seminar on site; 24 registered students dropped out prior to arrival. Third, the qualitative dataset comprised those who participated in the hackathon activities and produced hackathon data (*n* = 321); non-participation after arrival was due to illness, refusal, late arrival, or missing consent/assent. Baseline demographics are reported for the full on-site cohort (*n* = 336) to characterize the seminar population, whereas all qualitative analyses are based on the hackathon corpus (*n* = 321), as documented in the participant flow chart (Figure 1). Eligibility for study inclusion required timely arrival and completion of consent/assent procedures. For minors, written parental consent and adolescent assent were obtained; participation and use of hackathon materials for research were voluntary. Ethical approval was granted by the ethics committee of the Medical School Hamburg (MSH Hamburg; approval ID: reference no. MSH-2023/217). Data were anonymized prior to analysis and stored on a secure university server with access restricted to the study team. Photographic documentation was limited to non-identifiable artifacts (flipcharts, posters, written outlines) and excluded faces or other direct identifiers. Audio was recorded during plenary presentations. Because the hackathon addressed mental health topics with adolescents, a safeguarding procedure was in place. Participants were informed they could skip activities or withdraw at any time without negative consequences. Welfare structures within the seminar and designated safeguarding leads were available on site. Any acute distress, self-harm disclosure, or risk to self/others would have triggered immediate referral to safeguarding leads and, if required, local emergency services, following the seminar’s safeguarding procedures. The hackathon was embedded as a dedicated seminar day and ran from noon to noon (approximately 24 h). Participants worked in pre-assigned teams (typically 6–8 adolescents). Team allocation was carried out by the seminar organization using a partially randomized approach informed by skill-track considerations; teams were not intentionally balanced by country or gender beyond the randomization component.

The hackathon followed a staged pitching-competition format. Round 1 involved short team pitches delivered in smaller peer settings with peer voting; 12 teams advanced to Round 2, which involved jury evaluation, and four teams advanced to the final round held in plenary. The winning team received structured mentoring support and a EUR 500 seed grant provided by YFU. The incentive was part of the educational program and not conditional on research participation. Three primary qualitative data sources were generated. First, each team completed a structured written project outline using a standard template (for details about the hackathon procedure material and supporting documents, see Supplementary Materials S2), including a selected problem scenario, target group, project title, aims, project description, implementation plan, timeline, resource needs (people, organizational support, facilities, materials, technical resources), a brief budget rationale, anticipated barriers, and ideas for evaluation. Second, teams delivered oral pitches following shared pitching guidelines (for pitching guidelines, see Supplementary Materials S2), specifying at minimum the problem selected, goals, core activities, implementation approach, and a call to action describing how the prize would be used. Third, teams produced visual artifacts (posters/flipcharts, prototypes, or sketches) that were photographed and linked to the corresponding project files. Attendance was monitored by seminar staff and volunteers, but individual-level participation quality could not be reliably assessed within the collaborative format. A pre-seminar screening questionnaire (for details, see Supplementary Materials S1) informed the development of the hackathon’s problem scenarios. The questionnaire included demographic items (age, gender, country of origin) and check-all-that-apply items on exposure to mental health-related phenomena during and outside the exchange year (e.g., depression, anxiety, suicidality, loneliness, discrimination, prejudice, bullying, self-esteem and body image concerns, societal appearance ideals, homesickness, and digital well-being), plus a free-text field for additional experiences. The project team aggregated questionnaire insights into nine standardized problem scenarios framed as school-based or youth-community situations to prompt feasible, student-led interventions (also see Supplementary Materials S2). These scenarios served as deductive anchors for analysis and as structured prompts during hackathon work.

### 2.3. Reflexivity

The study team consisted of two authors (H.B. and A.-M.K.), both trained in qualitative methods. Both authors were present on site throughout the seminar and hackathon. AMK was additionally part of the seminar organizing team. To reduce expectancy effects and minimize winner bias, the authors did not participate in jury decisions or the formal evaluation of pitches. Coding was conducted blinded to whether teams advanced to later rounds. Audio recordings were transcribed verbatim by H.B. and A.-M.K. Each transcript was cross-checked by the other author against the original audio to improve accuracy and completeness. Volunteer notes used for facilitation were not treated as primary analytic data. No formal reflexive journaling was conducted; instead, transparency and reflexive control were supported through explicit code definitions, memoing in MAXQDA 26, iterative codebook revisions, and a documented consensus process. Stakeholder validation was conducted at the level of summary interpretations. YFU organizers reviewed the emerging thematic outputs for plausibility and contextual fit without re-contacting individual adolescents. These procedures were documented to strengthen transparency on researcher roles, reflexivity, and analytic credibility in line with qualitative reporting guidance [32,44].

### 2.4. Data Analysis

Given the interpretive and iterative nature of the analysis, formal intercoder reliability coefficients were not calculated. Instead, consistency was ensured through calibration coding, shared code definitions, and consensus-based resolution of discrepancies. defined, with no new subcategories emerging in later stages. All data sources (digitized project outlines, pitch transcripts, and linked images of flipcharts/posters) (see Supplementary Materials S4) were anonymized and imported into MAXQDA for management and analysis. The analytic unit was the team project as the case, with meaning-bearing segments coded within each case (e.g., statements describing the problem, intervention components, resources, barriers, and evaluation ideas). Coding followed the staged logic of content-structuring qualitative content analysis according to Kuckartz: (1) initial familiarization and case-oriented text work (including analytic memos and brief case summaries), (2) development of a preliminary main-category system aligned to the research questions and the predefined paths and problems, (3) first-cycle coding of the full corpus with these main categories, (4) inductive development of subcategories grounded in the material, (5) second-cycle coding using the refined category system, (6) simple and complex analyses (within- and across-case comparisons and relational analyses across dimensions), and (7) systematic documentation of analytic decisions and outputs (audit trail) [42,43]. To support interpretive consistency, both coders jointly coded two projects during a calibration phase to align segment boundaries, clarify category definitions, and harmonize coding granularity. The remaining corpus was divided for independent coding. Ambiguous segments were flagged and adjudicated in regular consensus meetings; code definitions and boundary rules were revised when required. Intercoder agreement coefficients were not computed; instead, credibility was pursued through calibration coding, explicit and evolving codebook definitions, and consensus-based reconciliation. Analytical saturation was operationalized pragmatically as codebook stability at the level of thematic structure: during late-stage coding, new subcategories were no longer required, and revisions were limited to clarifications of existing definitions rather than the introduction of substantively new themes. The final coding system (see Table 1) comprised three analytic dimensions aligned to the study aims: (i) Content/problem framing (what mental health-related issue and context the project addresses), (ii) Solutions/intervention approach (what type of solution is proposed and at what level), and (iii) Implementation (how feasibility, resources, barriers, and evaluation are conceptualized). This implementation dimension was conceptually aligned with implementation-strategy reporting recommendations, which emphasize the need to specify the actions, actors, targets, resources, and conditions required to move interventions into practice [45].

In addition to qualitative interpretation, coded data were used to generate descriptive visualizations, including heatmaps, co-occurrence networks, and alluvial diagrams. These illustrated patterns were used across projects and advancement stages. Because projects did not change in content across competition rounds, analyses were conducted as survivor-subset summaries, comparing thematic distributions across advancing groups. These summaries describe compositional differences between groups and do not reflect within-project development. Quantitative outputs were used for pattern illustration and triangulation rather than causal inference [46,47,48,49,50,51].

## 3. Results

### 3.1. Participant Characteristics and Mobility

A total of 336 adolescents (age range 15–19 years) participated in the hackathon (see Table 2). Overall, 234 participants were female (69.9%) and 102 were male (30.1%), with a mean age of 16.87 years (SD = 0.83). Participants were distributed unevenly across competition rounds: 321 (95.5%) were in Round 1, 90 (26.7%) in Round 2, 30 (8.9%) in Round 3, and 8 (2.4%) in Round 4 (see Table 2). Gender composition and mean age differed modestly across rounds. The descriptive statistics in this section characterize the full seminar cohort, whereas the alluvial and threshold-based summaries below describe the thematic composition of projects within teams that reached specific advancement stages. Participant characteristics should therefore be interpreted against the full on-site cohort (*n* = 336), while the subsequent analytic summaries refer to advancing subsets of projects.

**Figure 2:**
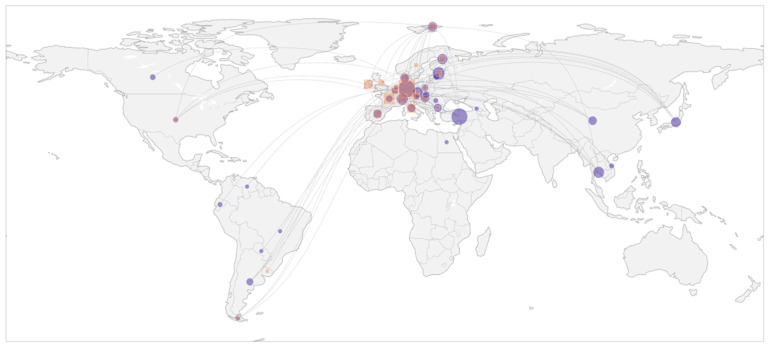
Connection map of home-host corridors with international mobility patterns. Legend: Violet circles = home countries; orange squares = host countries (for an interactive HTML graphic, see Supplementary Materials S5).

Participants traveled from 34 home countries to 22 host countries (see Figure 2), forming 143 distinct home–host corridors. Host placements were concentrated in a small number of destinations. Germany hosted 115/336 participants (34.2%), followed by France (42/336; 12.5%), Italy (26/336; 7.7%), Ireland (25/336; 7.4%), and Switzerland (23/336; 6.8%). On the sending side, the largest home-country groups were Germany (50/336; 14.9%) and Turkey (48/336; 14.3%), followed by Estonia (24/336; 7.1%), Switzerland (21/336; 6.3%), and Thailand (19/336; 5.7%). Despite the high number of unique travel corridors, movements were partially dominated by a small set of routes. The single largest corridor was Turkey to Germany (*n* = 32; 9.5% of all movements), followed by Germany to Ireland (*n* = 16; 4.8%) and Thailand to Germany (*n* = 10; 3.0%). The top 10 corridors accounted for 33.0% of all movements, and the top 20 corridors accounted for 45.8%, indicating a long-tailed structure in which many corridors were represented by only one or a few participants. Overall, the mobility pattern can be described as highly dispersed, with a small number of routes accounting for many participants and numerous additional corridors represented by only one or a few cases (for a detailed table with anonymized participant data, see Supplementary Materials S4).

### 3.2. Description of the Qualitative Corpus

The analytic corpus comprised 43 projects coded in MAXQDA across three domains: Content (problem framing), Solutions (intervention approach), and Implementation (feasibility and delivery) (for detail on all project transcripts, see Supplementary Materials S4). In total, 480 coded segments were identified: Implementation 214 (44.6%), Solutions 165 (34.4%), and Content 101 (21.0%).

**Figure 3:**
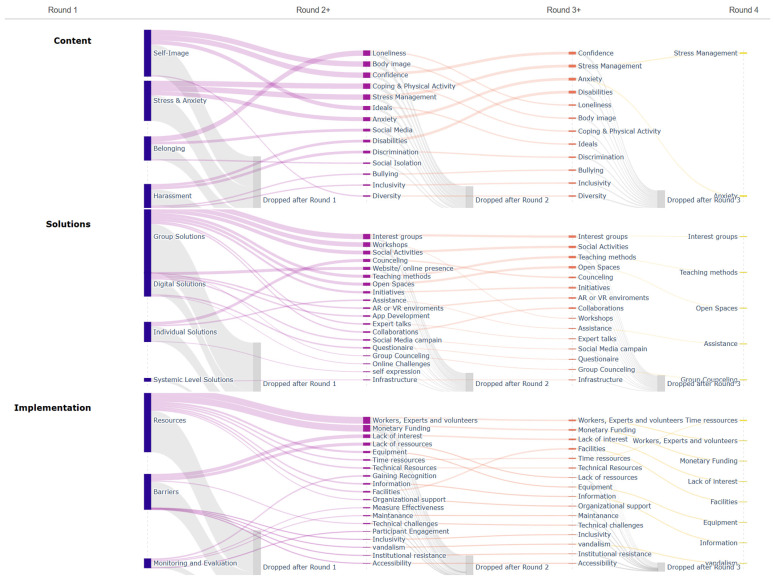
Alluvial diagram of the coding across hackathon rounds (for interactive HTML graphic, see Supplementary Materials S6). Legend: grey = dropouts; violet = made it to round 2; orange = made it to the finals, yellow = winning team (Black Albatros).

Nearly all projects contained material across the full analytic structure (43/43 projects included Content and Solutions; 42/43 included Implementation). At the project level, coded segments ranged from 5 to 23 (median 11), and projects varied in the breadth of level-2 codes (5–20, median 9), indicating substantial heterogeneity in how narrowly or broadly projects articulated their proposals. The importance of physical activity for mental health topics was already visible at the descriptive level. Some teams positioned movement as part of the solution itself, for example, through sports, hobby groups, workouts, and outdoor or shared activities, whereas others used activity more indirectly as a context for connection and confidence-building. The projects discussed in the analytic material illustrate this range (for detailed information about all projects, see Supplementary Materials S4): the group Black Bees linked belonging to shared games and sports, Pink Bee combined self-care with nutrition and physical activity, and Blue Goats coupled app-based support with workouts, habit tracking, and community events. Together, these examples suggest that adolescents often treat physical activity less as a standalone endpoint and more as a practical, socially embedded mechanism for improving well-being [8,10,11]. The three alluvial diagrams (see Figure 3) summarize how coded material is distributed across domains and code levels when projects are stratified by achieved rounds (Round 1 shown at higher-order clusters; later rounds at the level-2 category resolution).

Importantly, these diagrams describe the composition of projects belonging to teams that reached each round threshold, not additional content created in later rounds. Across the full set of coded material (Round-1 threshold), Implementation was the largest domain (44.6%), followed by Solutions (34.4%) and Content (21.0%), indicating that projects contained proportionally more feasibility and delivery-related statements than problem descriptions. When restricting to projects that advanced further, the domain composition remained broadly similar from Round 1 to Round 2 (Implementation 71/162; 43.8%, Solutions 55/162; 34.0%, Content 36/162; 22.2%), while the Round-3 threshold showed a relative increase in Solutions (Solutions 27/69; 39.1% vs. Implementation 26/69; 37.7%).

In the Round-4 threshold, coded material was dominated by Implementation (9/16; 56.2%), with a comparatively small Content component (2/16; 12.5%), reflecting a more compact problem-framing footprint alongside a larger feasibility footprint within the finalist subset. At the Content level-1 clusters (Round 1), problem framings concentrated in four higher-order areas: Self-Image (35/101; 34.7%), Stress & Anxiety (30/101; 29.7%), Belonging (18/101; 17.8%), and Harassment (18/101; 17.8%). At level-2 resolution, the most frequent categories were Confidence (19 segments; 13 projects) and Loneliness (11; 10 projects), followed by Anxiety (10; 7 projects) and Coping & Physical Activity (10; 6 projects); Bullying appeared in 7 segments across 7 projects. In later-round subsets, Content breadth narrowed: within the Round-4 threshold, Content was confined to Stress & Anxiety (specifically Anxiety and Stress Management, 1 segment each). Solutions were predominantly articulated as group-oriented formats. At level-1, Group Solutions accounted for 100/165 (60.6%), compared with Digital Solutions (33/165; 20.0%), Individual Solutions (27/165; 16.4%), and Systemic-level solutions (5/165; 3.0%). At level-2, the most common solution types were Social activities (19 segments; 13 projects), Workshops (18; 15 projects), and Interest groups (16; 13 projects), indicating a concentration on low-threshold, collective delivery modes across the corpus. Implementation content was dominated by resource planning and constraints. At level-1, Resources accounted for 125/214 (58.4%), Barriers for 70/214 (32.7%), and Monitoring/Evaluation for 19/214 (8.9%).

At level-2, the most widespread categories were Monetary funding (42 segments; 37 projects) and Workers/experts/volunteers (43; 34 projects), underscoring that staffing and financing were referenced across the majority of projects. Barrier statements were most frequently linked to lack of interest (26 segments; 25 projects) and lack of resources (18; 18 projects), indicating that feasibility considerations commonly included both motivational and material constraints (For details about Coding, see the full qualitative MAXQDA data corpus in Supplementary Materials S8).

### 3.3. Content–Solutions Linkage Patterns

**Figure 4:**
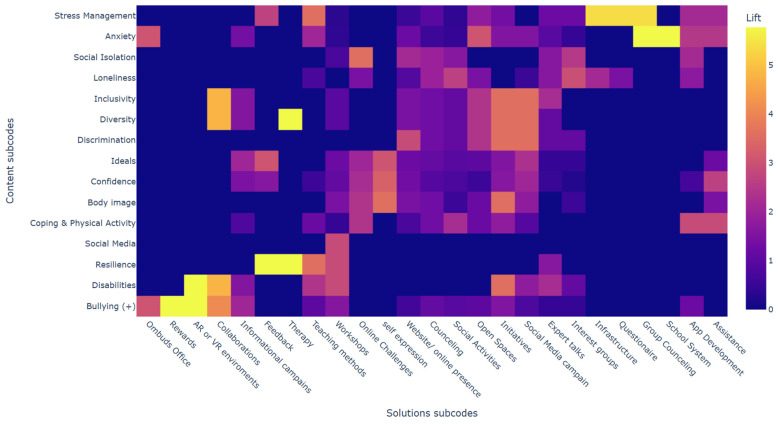
Content-Solutions association heatmap.

The Content-Solutions association heatmap (see Figure 4) summarizes co-occurrence regularities at the project level using counts and association metrics. Several concentrated pairings emerged. For projects coded for Loneliness (10 projects), the most consistent accompanying solution types were Interest groups (9/10; P(S|C) = 0.90) and Social activities (8/10; P(S|C) = 0.80). Given the base rate of Interest groups across the corpus (13/43 projects), the conditional concentration for Loneliness corresponded to a lift of approximately 2.98, indicating that Interest groups co-occurred with Loneliness substantially more often than expected from prevalence alone. A similar, though smaller-base, pattern was visible for Social isolation, where Interest groups appeared in 3/4 relevant projects (P(S|C) = 0.75; lift approximately 2.48). Stress-related content categories showed fewer but more selective links; for example, among Stress management projects (8), Questionnaire appeared in 3/8, which was notable because Questionnaire occurred in only 3 projects overall (yielding a high lift of approximately 5.38).

### 3.4. Solution and Implementation Strategies

**Figure 5:**
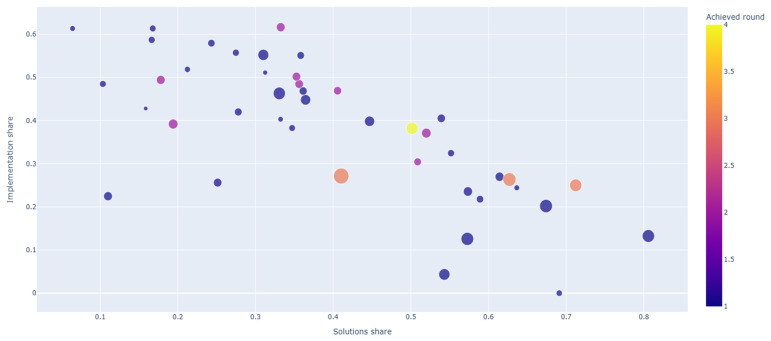
Solutions and implementation strategy map (for interactive HTML graphic, see Supplementary Materials S7). Legend: circle size = Total of coded segments in both categories.

The strategy map (see Figure 5) positions projects by their relative emphasis on Solutions and Implementation elaboration (based on coded span rather than segment counts). Across all projects, the median coded span was 378 for Solutions and 312 for Implementation, with wide ranges for both (Solutions 142–2232; Implementation 0–798).

Later-round projects tended to occupy regions characterized by higher implementation span: median Implementation span increased from 226 (Round 1) to 398.5 (Round 2), 515 (Round 3), and 609 (Round 4). The association between achieved round and Implementation span was moderate and monotonic (Spearman ρ = 0.54). Solutions span also increased across rounds, but less consistently (Spearman ρ = 0.29), while Content span showed little relationship with achieved rounds (Spearman ρ = 0.11). In addition, there is a non-linear shift in emphasis across competitive outcomes. The Round-4 winner is positioned near the center of the Solutions–Implementation space, reflecting a comparatively balanced elaboration of both domains rather than an extreme focus on either. In contrast, the Round-3-excluded projects cluster toward a solutions-heavy profile (higher relative Solutions elaboration with comparatively less Implementation detail), whereas the Round-2-excluded projects tend to show the opposite pattern: implementation-heavy profiles with more extensive feasibility and resourcing detail relative to their solution specification.

## 4. Discussion

This study examined which mental health-related problems internationally mobile adolescents prioritize and how they translated these concerns into solution concepts and implementation considerations within a structured, time-bounded co-creation format. Across the corpus, adolescents generated contextually grounded ideas and linked them to concrete feasibility considerations. Using a MAXQDA-based, content-structuring qualitative content analysis of 43 hackathon projects, we provide a descriptive map of the problem framings adolescents selected, the solution modalities they proposed, and the implementation issues they considered. More broadly, the findings add to emerging work showing that participatory youth mental health design can generate contextually grounded intervention concepts, while implementation-oriented analysis helps explain why some ideas appear more actionable than others [23,32,33,34,46,47,48,49,50,51,52].

First, participants’ problem framings converged into four higher-order clusters (Self-Image, Stress and Anxiety, Belonging, and Harassment) despite a highly international sample with 143 home-host corridors. At the same time, the subcategory level remained heterogeneous, indicating that teams rarely positioned mental health as a single-issue topic. This pattern mirrors the broader adolescent mental health literature, where anxiety-related distress, self-evaluative concerns, loneliness, and peer victimization are prominent sources of burden [4,5,6]. The narrowing of content breadth in later-round subsets should be interpreted cautiously, because projects were structured around primary problem scenarios and later-round analyses describe survivor subsets rather than iterative refinement of the same projects. Reframed against the Lubans et al. model, the strongest overlap with that framework lies mainly in psychosocial pathways such as self-perceptions, social connectedness, and coping-related processes. Importantly, the present material does not suggest that physical activity was the dominant logic across all projects; rather, movement-related elements appeared in some projects as one possible context through which social connection, confidence, or stress regulation might be supported [8,9,10,11,52,53].

Second, solution proposals converged more strongly than problem framings. Solutions were predominantly group-oriented (60.6% of solution segments), with common modalities centered on low-threshold, collectively organized formats such as social activities, workshops, and interest groups. This pattern is consistent with school- and community-based prevention approaches that target social functioning, coping, and peer processes [7,19,20]. In the qualitative corpus, many teams operationalized these ideas through practical, school-compatible formats. Several proposals combined peer exchange, mentoring, shared activities, digital communication channels, or light-touch movement elements. Taken together, the data suggest that adolescents primarily framed support in social and relational terms, even when some projects also incorporated sport, exercise, or hobby-based activity [22,25,53,54,55].

Third, cross-domain mapping indicated that adolescents did not apply generic solutions across all problem frames but developed recurring problem-solution pairings. Loneliness was disproportionately linked to interest groups and social activities, while social isolation showed a similar, smaller-base alignment with interest groups. Stress management was selectively linked to monitoring-oriented elements, and anxiety was paired with retreat-type solutions such as protected or calming spaces. These patterns indicate that teams produced recurring matches between specific concerns and specific formats rather than a uniform set of intervention ideas, which is a central feature of participatory design processes [29,31]. System-level solutions were rarely proposed, which may indicate that adolescents perceived structural change as more distant from their immediate sphere of influence. Where movement-related ideas appeared, they were typically embedded within broader social or routine-oriented formats rather than presented as standalone interventions [12,16,17,18].

Implementation considerations constituted the largest share of the coded corpus (44.6% of all segments), and feasibility discussions were dominated by resource planning and constraints. Funding and human resources were near-ubiquitous across projects, and barriers frequently referenced both motivational and material constraints. These findings align with implementation and school mental health literature showing that staffing, organizational support, contextual fit, and sustainment planning often shape whether interventions are adopted and maintained in real-world settings [29,35].

The strategy map adds a descriptive, performance-linked layer: implementation span increased with the achieved round, whereas solution span showed a weaker association. This pattern should not be interpreted as proof that implementation detail was the single driving force of project progression. Rather, within this specific competitive hackathon setting, projects that advanced further tended to combine a clear idea with more developed feasibility considerations. One plausible explanation is that such projects were easier for peers and jurors to evaluate because they reduced ambiguity and signaled readiness for practical application. At the same time, the competitive and time-constrained format may have favored ideas that were especially easy to communicate and appeared feasible, thereby shaping which projects progressed.

These findings contribute to current debates on how to generate prevention approaches that are both youth-aligned and implementable. Reviews of adolescent mental health interventions consistently report modest average effects and strong context dependence, underscoring the importance of contextual fit and delivery conditions [7,30,35]. Within that landscape, social hackathons may offer a structured way to surface adolescents’ priority concerns together with the delivery logics and practical constraints they perceive as relevant. In practical terms, schools or youth programs could use hackathon-style workshops to identify locally salient mental health challenges, co-develop low-threshold support ideas, and then refine the most promising concepts with staff, facilitators, or community partners before piloting them.

Participation in the hackathon occurred within the broader Youth Empowerment Seminar, and some participants may have been less intrinsically motivated to engage fully with the task. In addition, participants were assigned to internationally mixed groups that brought together adolescents from different countries and school systems. This diversity likely enriched the discussions, but it may also have encouraged broader, hybrid solution logics rather than highly context-specific interventions tailored to one local setting. Because students returned to different educational contexts after the seminar, the transferability of any single proposal to a typical school setting should be considered with caution.

Several limitations delimit inference. The sample reflects a voluntary exchange-student seminar cohort and may not represent adolescents without international mobility experiences or those in less resourced contexts. The hackathon format was time-bounded and competition-based; peer voting and jury selection may have shaped what was articulated and rewarded, and advancement is not a measure of intervention effectiveness. Round-based analyses therefore describe compositional differences among advancing projects rather than iterative improvement within unchanged teams. Finally, coded segments capture thematic emphasis within the available artifacts but do not reflect implementation outcomes, acceptability, or clinical impact. Future research should translate frequently occurring problem-solution pairings into prototyped interventions and evaluate them in staged designs, including feasibility pilots, assessments of acceptability and adoption conditions, and, where appropriate, effectiveness trials. Given the prominence of implementation-related considerations, early involvement of schools and organizational stakeholders appears particularly important [31,37,38].

In summary, the hackathon outputs show a convergent set of adolescent-prioritized mental health problem clusters within a highly international youth cohort, a strong preference for low-threshold social solution modalities, and a clear emphasis on feasibility considerations. Across the corpus, movement, sport, and hobby-based activities were usually framed as supportive contexts within broader social or psychosocial solution logics rather than as isolated endpoints in themselves. Together, these findings provide a data-grounded basis for selecting candidate components for subsequent prototyping and evaluation while preserving the adolescent perspective as a central design resource.

## 5. Conclusions

This study shows that a social hackathon can function as a feasible participatory research format for eliciting adolescent mental health priorities, solution ideas, and implementation considerations within a school-related prevention context. Across a highly international exchange student sample, adolescents prioritized topics around self-image, stress and anxiety, belonging, and harassment, while favoring social, peer-oriented, and low-threshold solution logics. Some proposals also incorporated movement- or sport-related elements, but these were typically embedded within broader psychosocial and implementation-oriented ideas. Projects that advanced further tended to present more explicit staffing, funding, and feasibility planning, although this pattern should be interpreted descriptively rather than causally. Future work should combine participatory design with explicit implementation planning, feasibility testing in real-world school settings, and careful adaptation to local contexts.

## Figures and Tables

**Table 1:** Final coding system. (For details, see full MAXQDA project in Supplementary Materials S8.)

Analytic Dimension	Level-1 Category	Level-2 Codes
Content (problem framing)	Stress & Anxiety	Anxiety; Stress Management; Resilience; Coping & Physical Activity
	Self-Image	Confidence; Body image; Diversity; Ideals
	Belonging	Social Media; Social Isolation; Loneliness
	Harassment	Inclusivity; Disabilities; Discrimination; Bullying
Solutions (intervention approach)	Individual Solutions	Self-expression; Counseling; Assistance; Feedback; Ombuds Office; Therapy; Rewards
	Digital Solutions	AR or VR environments; Website/online presence; Social Media campaign; Online Challenges; App Development; Questionnaire
	Group Level Solutions	Social Activities; Workshops; Expert talks; Interest groups; Informational campaigns; Initiatives; Open Spaces; Teaching methods; Group Counseling; Collaborations
	Systemic Level Solutions	Infrastructure; School System
Implementation	Resources	Organizational support; Equipment; Workers, Experts and volunteers; Monetary Funding; Facilities; Technical Resources; Information; Time resources
	Barriers	Vandalism; Competition; Lack of interest; Lack of resources; Technical challenges; Institutional resistance; Accessibility; Parental control; Inclusivity
	Monitoring/Evaluation	Measure Effectiveness; Participant Engagement; Maintenance; Gaining Recognition

**Table 2:** Participant characteristics on site and per hackathon round.

Characteristic	Participants on Site (N = 336)	Round 1 (N = 321)	Round 2 (N = 90)	Round 3 (N = 30)	Round 4 (N = 8)
Female, *n* (%)	234 (69.9%)	223 (69.5%)	62 (68.9%)	23 (76.7%)	7 (87.5%)
Male, *n* (%)	102 (30.1%)	98 (30.5%)	28 (31.1%)	7 (23.3%)	1 (12.5%)
Age, M (SD)	16.87 (0.83)	16.86 (0.83)	16.98 (0.82)	17.10 (0.88)	17.12 (0.83)

## Data Availability

All supporting documents and anonymized qualitative materials, including the full MAXQDA dataset analyzed in this study, are attached as Supplementary Material.

## Supplementary Materials

The following supporting information can be downloaded at: https://www.mdpi.com/article/10.3390/healthcare14101315/s1, The Supplementary Files include (S1) the pre-screening survey material, (S2) the hackathon procedure material and supporting documents, (S3) a table with detailed anonymized participant characteristics, (S4) all project transcripts, (S5–S7) interactive HTML graphics for all main findings, and (S8) the full qualitative MAXQDA data corpus.
