# Supplementary material for: Co-Creation of Mental Health Intervention for Adolescents: A Social Hackathon Approach
Source: Healthcare (Basel). 2026 May 12;14(10):1315. doi: 10.3390/healthcare14101315 (PMC13205654; doi:10.3390/healthcare14101315)
Supplement: Supplementary file 1 [file healthcare-14-01315-s001.zip › 1. Pre-Screening Survey material.pdf]

Pre-Screening Questionnaire

YES 2023 - Building resilitent minds - From Idea to Impact

Dear YFU Exchange Student,

The Youth Empowerment Seminar is the biggest event in YFU, organised entirely by YFU Volunteers for you! More details about our event here: https://www.yfu-yes.org/

This year, the topic is **mental health!**

The participants of the YES will have the chance to design a project which will takle a mental health issue in their local community. However we would like to hear from you, what are the main issues you or your friends are facing in terms of mental health, in order to stirr the conversation in the right direction. We all know this is a very broad topic, so we want to stay close to your needs.

Thank you for taking 3 minutes to complete this questionnarie!

Follow us on Instagram: @yfuyes !

* Gibt eine erforderliche Frage an

1. What is your age? *

____

2. Whats your gender? *

Markieren Sie nur ein Oval.

- ( ) diverse
- ( ) female
- ( ) male

3. What is your country of origin? * Dropdown

Markieren Sie nur ein Oval.

- Argentina AR
- Australia AU
- Austria AT
- Azerbaijan AZ
- Belgium (Wallonia) BE
- Belgium (Flanders) BE
- Brazil BR
- Bulgaria BG
- Canada CA
- Chile CL
- China CN
- Colombia CO
- Costa Rica CR
- Czech Republic CZ
- Denmark DK
- Ecuador EC
- Estonia EE
- Finland FI
- France FR
- Germany DE
- Ghana GH
- Greece GR
- Hungary HU

- India IN
- Indonesia ID
- Italy IT
- Japan JP
- Kazakhstan KZ
- Latvia LV
- Liberia LR
- Lithuania LT
- Mexico MX
- Moldova MD
- Mongolia MN
- Netherlands NL
- Norway NO
- Paraguay PY
- Philippines PH
- Poland PL
- Romania RO
- Serbia RS
- Slovakia SK
- South Africa ZA
- South Korea KR
- Spain ES
- Sweden SE
- Switzerland CH

- Thailand TH
- Turkey TR
- Ukraine UA
- Uruguay UY
- USA US
- Venezuela VE
- Vietnam VN
- Other

4. During your time abroad, were you exposed to mental health problems? Please check which of the following phenomena you experienced. *

Wählen Sie alle zutreffenden Antworten aus.

- [ ] I didn't experience mental health issues
- [ ] Depression
- [ ] Anxiety
- [ ] Suicide
- [ ] Hate speech
- [ ] Social acceptance
- [ ] Loneliness
- [ ] Discrimination
- [ ] Prejudice
- [ ] Societal ideals relating to appearance
- [ ] Eating behavior
- [ ] Body image
- [ ] Self-esteem
- [ ] Bullying
- [ ] Digital well-being
- [ ] Homesickness
- [ ] Sonstiges: ______________________

5. Outside of your time abroad, were you exposed to mental health problems? Please check which of the following phenomena you experienced. *

Wählen Sie alle zutreffenden Antworten aus.

- [ ] I didn't experience mental health issues
- [ ] Depression
- [ ] Anxiety
- [ ] Suicide
- [ ] Hate speech
- [ ] Social acceptance
- [ ] Loneliness
- [ ] Discrimination
- [ ] Prejudice
- [ ] Societal ideals relating to appearance
- [ ] Eating behavior
- [ ] Body image
- [ ] Self-esteem
- [ ] Bullying
- [ ] Digital well-being
- [ ] Homesickness
- [ ] Sonstiges: ______________________

6. Please let us know about any other mental health problems you have expereinced during or outside your exchange year. *

____

____

____

____

____

Dieser Inhalt wurde nicht von Google erstellt und wird von Google auch nicht unterstützt.

Google Formulare

__Answers to Pre-Screening Questionnaire__

YES 2023 - Building resilitent minds - From Idea to Impact

59 Antworten

What is your age?

Kopieren

59 Antworten

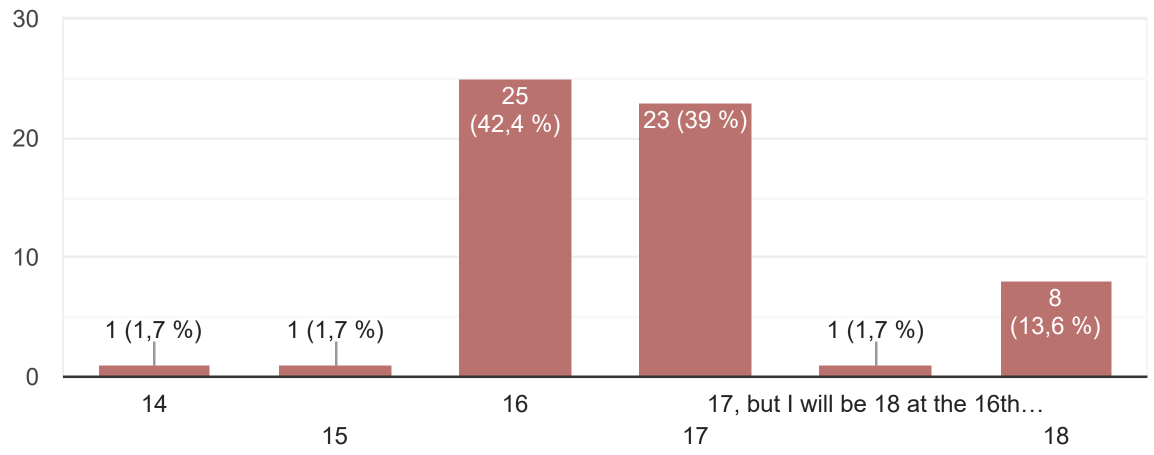

Whats your gender?

Kopieren

59 Antworten

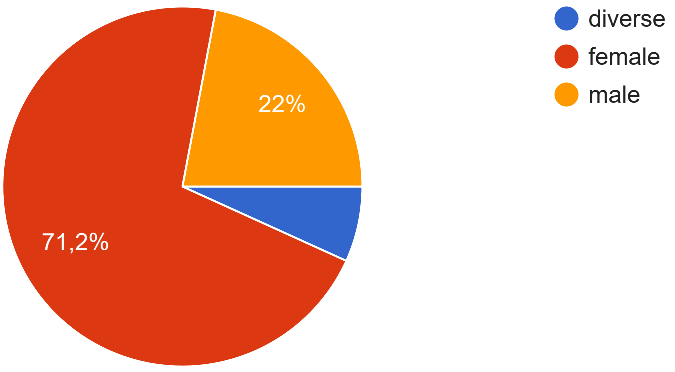

What is your country of origin?

Kopieren

59 Antworten

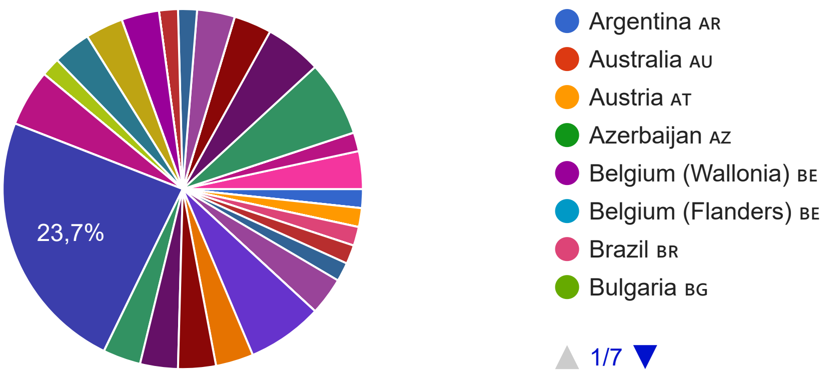

During your time abroad, were you exposed to mental health problems? Please check which of the following phenomena you experienced.

Kopieren

59 Antworten

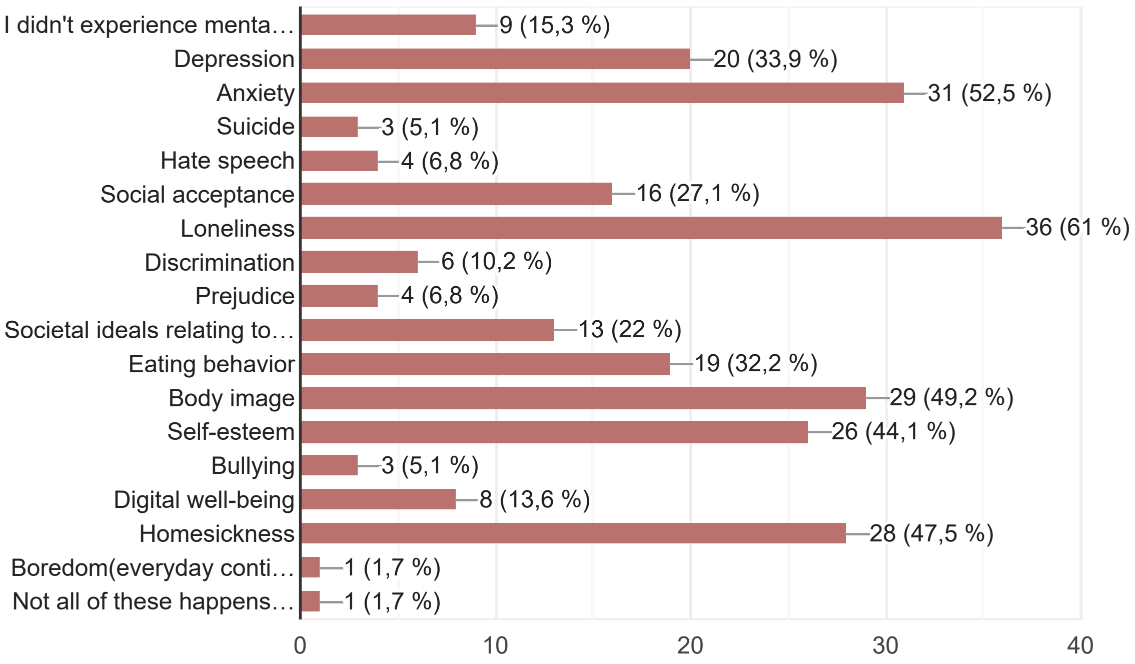

Outside of your time abroad, were you exposed to mental health problems? Please check which of the following phenomena you experienced.

Kopieren

59 Antworten

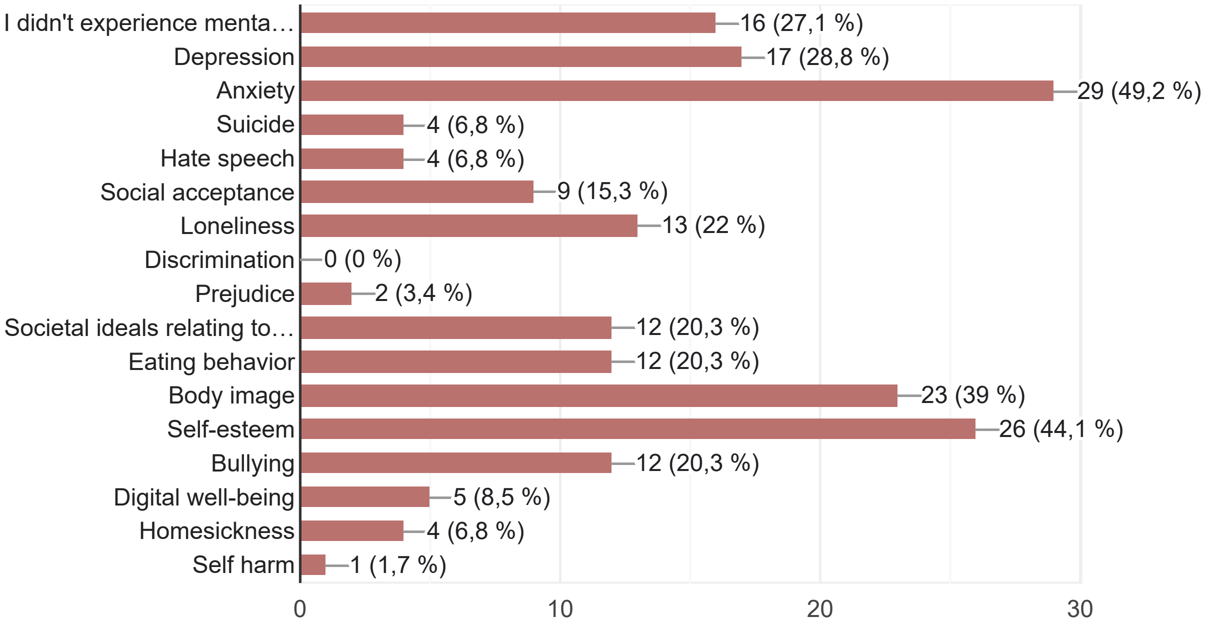

Please let us know about any other mental health problems you have expereinced during or outside your exchange year.

59 Antworten

/

.

-

None

(This is just a note): of the problems I've reported, only homesickness remains, all the others I've overcome and I'm fine with them.

It was hard to get up in the mornings, I felt like I was wasting my time which lead to anger and frustration, boredom

The way social media and digitalisation had an impact on the topics named, I've also struggled with addiction such as nicotine which got worse during my year abroad

Not good enough

i dont have

A lot of mentaly tough periods

Nothing

none

There were times where i was so anxious that i had the feeling of needing to throw up because some situations stressed me so much , this was during my exchange year

Nothing else

Problems with self harm

not wanting to go back to home country, social anxiety, simple confusion about life and future, overthinking

I havent

Ok

No other

I had a down phase during my exchange, I would not call it a depression, because of an heavy illness of my mother at home and because of an breakup during the exchange.

Communication problems

Insecure

Climate “anxiety” (don’t like to use this term as it’s a logical fear, but anyway) and a general societal worry and fear. I really think it’s so important that we share and talk about all of the feelings the climate and biodiversity crisis impose on us. Speaking of mental health for the youth, I don’t think it can be excluded. For me it has been even more difficult on exchange, as I haven’t been able to do a lot of activism and I don’t have a community to share it with. It would be wonderful if this will be discussed:)

No other problems

abandonment issues

I did not have any other mental health problems.

N/A

All named above

I used to feel very anxious here sometimes, because of the pressure to learning the language as fast as possible and making friends as fast as possible. This anxiety stopped when I started taking my time for each thing and respecting my limits, thought.

—-

Suicidal thoughts because of bullying

social anxiety

I don’t understand this question, I think I covered most of it in the ones before

I always feel like I'm am apart from the community and before the exchange year I don't like that but now I am ok with this.

I sometimes feel hopeless if the environment I am in doesn't have a value I agree with but I am learning to deal with this.

I used to be very anxious about my future pass and afraid any tiny movement will make a negative change of it.

And I wanted everything was perfect that leads to procrastination and also lots of self-anxiety.

I sometimes just wanted to stay in my comfort zone or just did what I was familiar with and also wanted to finish my plan. If I fail I would feel bad about myself for a while.

That's all
I love this topic, looking forward to it
Thank you :)

I’ve changed 4 times family and with my second family I’ve experienced so much mental violence and the family made me feel always wrong unsure about everything and I’ve had so bad experiences with them.

Thoughts of losing weight through eating disorders

I don't know if it's a mental health problems. But that I realized I can't change how life is going. Like diseases of family and friends.

I didn't experienced another mental health problem

Kein Problem

Trauma, eating disorder, panic attacks

i haven’t had any mental health issues

I wouldn't be able to fit this in any of the previous options, but I think it's also important for me to mention that, from some months after I started to go to school here in Switzerland until today, things have just went worse and they don't seem to show any hint of possible change. I don't really feel that I have a place there anymore and I'm never able to do much in there (on academic therms), that has been causing me lots of struggles to keep motivated, instead, with time my anxiety and energy have gotten much worse than I ever thought they could be. I have to be honest, but I might not be liking the experience at school here, I don't feel comfortable and happy anymore.

There weren't any other

nothing else

Loss of motivation

Dieser Inhalt wurde nicht von Google erstellt und wird von Google auch nicht unterstützt. - Eigentümer dieses Formulars kontaktieren - Nutzungsbedingungen - Datenschutzerklärung

Sieht dieses Formular verdächtig aus? Bericht

Google Formulare

Pre defined Paths and Problems

A. Self-esteem, body image issues, societal ideals related to appearance - How do we promote self esteem to encourage a more positive and healthier body image?

Problem A1: Social Media - How might we empower the youth to cultivate a healthier relationship with social media?

Meet Jane, a 16-year-old student at YFUVILLE High School. Like many of her peers, Jane often finds herself caught in the cycle of comparing her life to the carefully curated images on social media. The flawless appearances, glamorous lifestyles, and endless friendships showcased on platforms like Instagram and TikTok leave her feeling inadequate and frustrated. The pressure to conform to societal ideals and the constant exposure to these unrealistic standards have taken a toll on Jane's self-esteem and overall well-being.

The impact of social media on young people's self-esteem and body image is a significant concern in YFUVILLE High School. Recognizing the need to address this issue, the school is calling for local projects that help students gain a better understanding of how social media operates and empower them to cultivate a healthier relationship with it. The aim is to reduce harmful comparison, alleviate feelings of failure and frustration, and promote a more positive and authentic sense of self.

Challenge:
Your team's challenge is to create a project that equips the students at YFUVILLE High School with the knowledge and skills to navigate social media responsibly while nurturing a positive self-esteem. Here are some key questions, that will help guide you when designing your project, which should support the students in developing a healthier perspective on social media and encourage a more positive and realistic body image:

1. How can you educate the students about the curated nature of social media and the manipulation techniques used to create an idealized image? Consider organizing workshops or interactive sessions that expose the students to behind-the-scenes aspects of social media and teach them how images can be edited and filtered.
2. What strategies can you implement to help the students develop a healthier perspective on social media, reducing the negative impact on their self-esteem and body image? Explore activities that promote self-acceptance, celebrate diverse body types, and challenge societal beauty standards.

3. How might you empower the students to critically analyze social media content, question unrealistic standards, and build resilience against harmful comparison? Foster discussions that encourage critical thinking, media literacy, and the importance of valuing individuality.
4. What resources, workshops, or interactive activities can you provide to support the students in building a positive relationship with social media while maintaining a healthy self-esteem? Offer resources that promote digital well-being, such as guidelines for mindful social media use and tips for fostering positive online interactions.
5. How can you collaborate with the local school, teachers, parents, and community members to create a comprehensive and sustainable program that addresses these challenges effectively? Engage stakeholders through partnerships, involve parents in awareness campaigns, and integrate the program into the school curriculum.

By addressing these questions and developing a comprehensive project, your team will empower the students at YFUVILLE High School to understand the influence of social media on self-esteem, develop a healthier body image, and navigate social media with confidence. Together, you can create a supportive and positive environment that encourages authenticity, self-acceptance, and a more balanced relationship with social media.

A. Self-esteem, body image issues, societal ideals related to appearance - How do we promote self esteem to encourage a more positive and healthier body image?

Problem A2: Societal ideals related to appearance - How can we support students in leaving behind harmful societal ideals and beauty standards?

In the town of YFUVILLE, the local high school has observed a growing concern among its students regarding appearance-based goals and a lack of focus on overall health and well-being. Many young people are influenced by societal pressures to conform to specific body standards, often at the expense of their mental and physical health. Many students feel immense pressure to conform to the societal ideals of appearance that surround them. If their appearance doesn't align with the narrow beauty standards promoted by society, they often feel excluded and judged as a result. This constant scrutiny has led to a negative impact on their body image, causing them to develop poor self-esteem. It has become crucial to shift the narrative and promote the importance of physical activity, nutritious eating habits, and self-care practices that support holistic well-being.

The impact of societal ideas related to appearance is a significant concern in YFUVILLE High School. Recognizing the need to address this issue, the school is calling for local projects that help students gain a better understanding of how these standards are harmful for society as a whole, and the youth in particular. The aim is to expose and raise awareness on how unrealistic beauty standards limit self-esteem, affect self-worth and create pressure to conform to societal beliefs in order to be accepted and valued by their peers.

<u>Challenge:</u>
Your team has been given the task of developing a project at the local high school that shifts the focus from appearance-based goals to a more comprehensive approach to health and well-being. The goal is to educate and empower students to prioritize physical activity, nutritious eating, and self-care practices that support both their mental and physical health. By doing so, the project aims to create a positive and sustainable impact on the students' overall well-being and self-perception. Here are some key questions, that will help guide you when designing your project:

1. How can we shift the focus from solely appearance-based goals to overall health and well-being? How can we promote the importance of physical activity, nutritious eating habits, and self-care practices that support mental and physical health?
2. How can you raise awareness among students about the negative consequences of solely focusing on appearance-based goals and the importance of holistic health and well-being?
3. What strategies can be implemented to promote physical activity among students, considering their diverse interests and abilities? How can you make it fun, engaging, and accessible to all?
4. How might you provide education on nutrition and promote nutritious eating habits, taking into account individual dietary preferences, cultural backgrounds, and budget constraints?
5. What self-care practices can be introduced and encouraged to support students' mental health and stress management? How can you make self-care an integral part of their daily routines?
6. How can you foster a supportive environment that encourages students to prioritize their overall health and well-being? How can you involve teachers, parents, and the broader school community in this effort?
7. How might you track and measure the impact of the project on students' attitudes, behaviors, and overall well-being? What indicators can be used to assess progress and success?

By addressing these questions, your team can develop a comprehensive project that shifts the focus from appearance-based goals to promoting overall health and well-being among the students. Through education, engagement, and the creation of a supportive environment, the high school in YFUVILLE can become a place where students prioritize their physical and mental health, embrace healthy habits, and develop a positive relationship with themselves and their bodies.

A. Self-esteem, body image issues, societal ideals related to appearance - How do we promote self esteem to encourage a more positive and healthier body image?

Problem A3: Self-worth, confidence and self-love - How can we contribute to strengthening a sense of self valoration in young people?

Meet Teo, a 17-year-old high school student who has been struggling with low self-worth and self-confidence. He often doubts his abilities, feels inadequate compared to his peers, and finds it challenging to express himself authentically. These feelings of inadequacy and dissatisfaction have permeated various aspects of Teo's life. Academically, he struggles to focus and perform to his full potential due to the constant distraction of negative self-talk. Socially, Teo finds it challenging to connect with his peers authentically. He often hides behind a façade, afraid that expressing himself genuinely will lead to rejection or ridicule. This fear of not being accepted as he truly is has created a barrier to forming meaningful relationships and participating fully in social activities.

Recognizing the importance of building self-worth and self-confidence in young people like Teo, YFUVILLE high school is looking for projects that foster a strong sense of self and empowers students to embrace their unique qualities. The goal is to implement initiatives that promote self-acceptance, raise awareness about self-love and support students like Teo to build confidence in who they are.

<u>Challenge:</u>
Your team's challenge is to develop a comprehensive project that promotes self-worth, self-love and self-confidence among young people. By addressing the following key questions, you can create a transformative program that empowers them to recognize their value and embrace their individuality:

1. How can we contribute to strengthening a sense of self-worth and self-confidence in young people?
2. How can you create a safe and inclusive environment where young people feel comfortable exploring and expressing their true selves?
3. What strategies can you implement to help young people recognize their strengths, talents, and unique qualities? How can we promote self-reflection, self-discovery, and celebration of personal achievements?

4. How might you encourage positive self-talk and develop strategies to challenge negative self-perceptions?
5. What resources and tools can you provide to support young people in building self-confidence?
6. How can you foster a culture of support and encouragement within the community?
7. How might you collaborate with schools, parents, and community organizations to create a sustainable program that reaches a broader audience?

By addressing these questions and developing a transformative program, your team can make a significant impact on young people's lives, empowering them to recognize their inherent worth, embrace their uniqueness, and develop the self-confidence to navigate life's challenges. Through fostering a positive self-image and a strong sense of self, they will be better equipped to pursue their dreams, overcome obstacles, and lead fulfilling lives.

B. Loneliness, isolation, lack of belonging - How do we reduce feelings of loneliness, isolation and lack of belonging?

Problem B1: Loneliness - How might we foster a culture of inclusivity and collaboration in schools?

Meet Sophie, a student at your local school (Harmony Middle School) who has been experiencing deep feelings of loneliness and distress in recent weeks. She finds it challenging to connect with her classmates as they seem to have no shared hobbies or topics to talk about. They show little interest in her and sometimes even make fun of her. Initially, Sophie attempted to reach out to them, but after facing rejection multiple times, she lost the courage to try again. She stopped attending activities she was invited to and displayed no interest, causing her classmates to stop including her in their plans.

The feelings of loneliness, isolation, and a lack of belonging have taken a toll on Jane's mental health and overall well-being. She has experienced a decline in academic performance, a decrease in participation in extracurricular activities, and a general decline in her morale as a student. Therefore, Harmony Middle School is searching for projects created by young people who can make this issue visible and offer a solution.

<u>Challenge:</u>
Your team has been tasked with creating a project that can help young people like Sophie integrate into their local community, find connections, and overcome their feelings of exclusion. The goal is to foster a culture of understanding, empathy, and effective communication among students. Here are some questions to guide your creation process:

1. How can you encourage the groups of students to be more inclusive and welcoming to those who tend to isolate themselves?
2. How can you raise awareness among the students about the negative impacts of isolation and the importance of inclusivity?
3. How can you create opportunities for students to find common points of interest and engage in activities that foster connection and friendship? What initiatives can you introduce to encourage shared hobbies, clubs, or projects that bring students together?
4. How can you facilitate communication and understanding between students who might appear different from one another?
5. How can you break down stereotypes and prejudices that hinder relationships? What strategies can be employed to promote open-mindedness and respect?

6. How can you leverage the knowledge and understanding gained from discussions about prejudices and stereotypes to bridge the differences between these young people? How can you create platforms or activities that facilitate dialogue, empathy, and the sharing of diverse perspectives?
7. How can you provide support and guidance to students like Sophie, who are experiencing loneliness and a lack of belonging?

By addressing these questions and developing a comprehensive project, your team can create a supportive and inclusive environment where young people like Sophie feel integrated, valued, and connected. Through awareness, activities promoting common interests, improved communication, and support mechanisms, your project can help bridge the gaps and foster a sense of belonging for all students within the local community.

B. Loneliness, isolation, lack of belonging - How do we reduce feelings of loneliness, isolation and lack of belonging?

Problem B2: Bullying - How can we empower students to stand up against isolating and harming others?

One of the main characteristics of Harmony Middle School is that students are very culturally diverse. They usually learn in harmony and have respect for each other. However, within this vibrant school community, a challenge persists - there are frequent incidents of bullying that result in the isolation of certain students. Bullying takes various forms, including verbal taunts, social exclusion, and online harassment, leaving victims feeling isolated, alone, and profoundly impacted. Research has shown that bullying can have severe consequences, leading to long-term negative effects on the mental health, academic performance, and overall well-being of victims.

The impact of bullying in the learning community of Harmony Middle School is a concern as it hinders growth, empowerment and collaboration. Therefore, the school is looking for local projects that equip students to stand up against bullying and support students in raising awareness about this.

Challenge:
Your team has been given the crucial task of developing a project at Harmony Middle School that empowers students to stand up against bullying and prevent the isolation of others. The goal is to create a school culture where kindness, empathy, and respect thrive, ensuring that no student experiences the harmful effects of bullying or feels isolated within the school community. By addressing the following key questions, you can create a transformative program:

1. How can you raise awareness among students about the prevalence and harmful effects of bullying, emphasizing the importance of preventing isolation?
2. What initiatives can you implement to educate students about different forms of bullying, including cyberbullying, and equip them with strategies to identify, report, and address incidents of bullying?
3. How might you empower students to become upstanders and intervene when they witness bullying, fostering a sense of responsibility for the well-being of their peers?
4. What resources can you provide to both victims and perpetrators of bullying, promoting empathy, personal growth, and digital citizenship?

5. How can you involve teachers, parents, and the wider school community in creating a collaborative effort to prevent bullying and isolation, fostering a safe, inclusive, and supportive environment for all students?

By addressing these questions, your team can develop a comprehensive project that not only raises awareness but also empowers students to take a stand against bullying, preventing the isolation of others. Through education, support, and community involvement, Harmony Middle School can become a place where every student thrives, demonstrating kindness, empathy, and a commitment to inclusivity, and where the harmful impact of bullying is mitigated.

B. Loneliness, isolation, lack of belonging - How do we reduce feelings of loneliness, isolation and lack of belonging?

Problem B3: Value in Diversity - How can we make our learning spaces more inclusive to neurodivergent people and people with disabilities?

At Harmony Middle School, a wide range of students with diverse learning needs and abilities come together to learn and grow. However, there is a pressing challenge - ensuring that the learning spaces are inclusive and accommodating for neurodivergent individuals and those with disabilities. Neurodivergent individuals have unique ways of thinking and processing information, such as those with autism spectrum disorders, ADHD, dyslexia, or other cognitive differences. People with disabilities have different conditions that require multiple ways of addressing them (accommodating spaces, having more facilities, adapting lessons and materials, etc.). It is crucial to create an environment that supports their learning styles and promotes their overall well-being.

The impact of understanding value in diversity, specially cognitive and physical diversity, is a pressing issue for your local school at Harmony. What is concerning is that teachers are not trained, buildings are not equipped and the system is not so supportive. This hinders growth, empowerment and development of these individuals.

Challenge:
Your team has been given the important task of developing a project at Harmony Middle School that focuses on making learning spaces more inclusive for neurodivergent individuals and people with disabilities. The goal is to create an environment that recognizes and celebrates neurodiversity, providing equal opportunities for all students to thrive and succeed. By addressing the following key questions, you can create a program that makes visible and equip students with the right tools:

1. How can you raise awareness and understanding among students, teachers, and staff about neurodiversity, helping them recognize and appreciate the different ways individuals may experience and process information?
2. What modifications and accommodations can be implemented in learning spaces to support the specific needs of neurodivergent students and people with disabilities? How can technology support you all in this quest?
3. How might you promote a culture of empathy and acceptance within the school community, fostering understanding and support for neurodivergent individuals?

4. What training and professional development opportunities can be provided to teachers and staff to enhance their knowledge and skills in creating inclusive learning environments?
5. How can you involve neurodivergent students themselves in shaping the learning spaces, seeking their input, and ensuring their voices are heard?
6. How can you involve students with disabilities? How can you promote their empowerment and support them to communicate their needs better?

By addressing these questions, your team can develop a comprehensive project that promotes inclusivity and accommodates the unique learning needs of neurodivergent individuals and people with disabilities. Through awareness, modifications, and a supportive school culture, Harmony Middle School can become a place where all students can thrive and reach their full potential.

C. Stress - How do we improve our relationship with stress?

Problem C1: School and stress - How can we make our school a less stress inducing place?

Welcome to Oakwood High, a bustling high school where students are all too familiar with the overwhelming pressure and stress that come with academic demands, social expectations, and extracurricular commitments. This is a very typical school: students often find themselves overwhelmed by a heavy workload, frequent exams, and the constant pressure to excel in order to secure a bright future. Additionally, social pressures to fit in, maintain high grades, and participate in numerous activities create a demanding atmosphere that can take a toll on students' mental and emotional well-being.

While the school administration may not fully recognize the extent of the problem, a group of proactive and motivated students have come together to address the issue head-on. They need support in creating an initiative, a project that help them make the issue visible, equip students and teachers with the right tools not only to handle stress but also to foster a less stressful atmosphere.

Challenge:
Support these students who have observed the toll that excessive pressure and stress take on the well-being of Oakwood High's student body. Determined to make a positive impact, your team embarks on a mission to develop a project that will transform the school into a less pressure-filled place. Your goal is to raise awareness, advocate for change, and create a supportive and nurturing environment that prioritizes student well-being. Use the following questions to guide your creative process:

1. How can you raise awareness among students about the detrimental effects of excessive pressure and stress on their mental health and overall well-being?

2. What initiatives can you implement to educate students about the consequences of discrimination and equip them with strategies to identify, report, and address it? What initiatives can you implement to provide tangible support for students who are grappling with overwhelming pressure?
3. How can you foster open and honest dialogue about the challenges students face within the school community?

4. What changes can you advocate for within the school environment to alleviate the excessive pressure and create a healthier atmosphere?
5. How can you collaborate with teachers and staff to ensure they understand and respond to students' needs regarding pressure and stress? Can you facilitate professional development sessions, provide resources, or foster an environment of empathy and support among the faculty?
6. How can you actively gather feedback and insights from students to continually improve the school's approach to addressing pressure and stress?

By addressing these questions and harnessing the collective power of determined students, your team can develop a comprehensive project that fosters a healthier and more supportive environment at Oakwood High. Through awareness, support systems, open dialogue, advocacy, and collaboration with both students and staff, you can pave the way for a school culture that values well-being, resilience, and overall student success.

C. Stress - How do we improve our relationship with stress?

Problem C2: Stress and discrimination - How can we reduce the stress triggered by discrimination in school?

Meet Alex, a 17-year-old student attending Oakwood High. Alex belongs to an ethnic minority group and has been facing discrimination and prejudice from some of their classmates. This discrimination not only creates a hostile and unwelcoming environment but also triggers immense stress and emotional turmoil for Alex.

Discrimination in schools is a pervasive issue that can have severe consequences for the well-being and academic performance of students. It is crucial to address this problem and create a school environment that promotes inclusivity, respect, and reduces the stress triggered by discrimination, which can end very tragically. Oakwood High acknowledges these situations and is looking for projects created by students, that can bring innovative solutions to the table.

Challenge:
Your team has been tasked with developing a project that focuses on reducing stress triggered by discrimination in Oakwood High. The goal is to create a supportive and inclusive school community that actively combats discrimination, fosters empathy, and provides resources to support students who experience discrimination-related stress. The following questions will help you create a solution to this problem:

1. How can you raise awareness about the impact of discrimination on students' mental health and overall well-being?
2. What strategies can you implement to promote empathy, understanding, and acceptance among students?
3. How can you provide support and resources to students who experience discrimination-related stress?
4. What policies and protocols can be implemented within the school to address instances of discrimination and ensure swift and fair responses?
5. How can you empower students to become active allies in the fight against discrimination?
6. How can you foster a positive and inclusive school culture that celebrates diversity and rejects discrimination?

By addressing these questions and developing a comprehensive project, your team can work towards reducing the stress triggered by discrimination in Oakwood High. Through raising awareness, fostering empathy, providing support, implementing policies, empowering students, and promoting an inclusive school culture, you can create an environment where all students feel safe, respected, and able to thrive academically and emotionally.

C. Stress - How do we improve our relationship with stress?

Problem C3: Stress and anxiety - How can we empower people with anxiety to speak up about it and claim space for their needs?

You might know Sam, a bright and talented student at Oakwood High. Sam is known for their exceptional academic achievements and active involvement in various extracurricular activities. However, behind their confident facade lies a constant battle with anxiety. Sam is often overwhelmed by worries and fears, making it difficult for them to fully engage in school life and express their needs. Sam is not alone in this struggle, as many other students within the school silently cope with anxiety, feeling unheard and misunderstood.

Anxiety, a common mental health condition, affects a significant number of individuals, including students, and can have a profound impact on their well-being and academic performance. It is crucial to create an environment that empowers individuals with anxiety to speak up, advocate for their needs, and claim space within the school community. The school is looking for local projects to support students with these pressing issues.

<u>Challenge:</u>
Your team has been assigned the task of developing a project that focuses on empowering people with anxiety to express themselves and seek support in Oakwood High. Your goal is to foster a culture of understanding, empathy, and inclusivity, where individuals with anxiety can openly discuss their experiences and access the necessary resources to thrive. These are some guiding questions that will help you create a comprehensive project:

1. How can you raise awareness about anxiety and its impact on individuals' lives among students, teachers, and staff?
2. What strategies can you implement to create a safe and non-judgmental space for individuals with anxiety to express their needs and concerns?
3. How can you empower individuals with anxiety to speak up and advocate for their needs within the school setting?
4. What resources and support systems can be implemented within the school to address the specific needs of individuals with anxiety?
5. How can you foster a culture of empathy and understanding within the school community to ensure that individuals with anxiety feel supported and accepted?
6. How can you collaborate with teachers, counselors, and administrators to create a comprehensive support network for individuals with anxiety? What strategies can you

think of to provide professional development opportunities for staff, establishing clear communication channels, and integrating mental health resources into the school's overall support framework?

By addressing these questions and developing a comprehensive project, your team can empower individuals with anxiety to speak up, claim space for their needs, and access the support they require to thrive at Oakwood High. Through awareness, understanding, and a proactive approach to mental health, you can create a school environment that fosters inclusivity, support, and overall well-being for all students.
