# Supplementary material for: Co-Creation of Mental Health Intervention for Adolescents: A Social Hackathon Approach
Source: Healthcare (Basel). 2026 May 12;14(10):1315. doi: 10.3390/healthcare14101315 (PMC13205654; doi:10.3390/healthcare14101315)
Supplement: Supplementary file 1 [file healthcare-14-01315-s001.zip › 2. Hackathon procedure material.pdf]

Pitching guidelines for students- how to craft the winning pitch?

Pitching format:

- You need to choose one problem from the ones given and create a solution/project that aims at helping your local communities develop mental resilience - it needs to be as tangible as possible.
- The project is a direct response to the problem and is applicable by students like you and in communities like the ones you move within (school, family, neighborhood)
- The pitch should be 5 minutes max!
- Any analogue visual aids are allowed (ie. flipcharts, prototypes, etc.). There will not be any projectors available for the presentations, but if you want to show a picture or an element on a tablet or laptop, this will be allowed.
- No restrictions on how many people should deliver the pitch
- The pitch has to include (this list is not chronological!):
  - Project name
  - Each participants name
  - Problem chosen
  - Target group
  - Defined project goals - what is the outcome they are trying to achieve
  - Description of the project
  - Implementation plan
  - Timeline
  - Call to action: What do you want the prize for?
- Remember to practice your pitch multiple times to ensure clarity, brevity, and effective communication of your project. Also, remember to answer the questions: why? For whom? What? How? By whom?
- Pitching Round 1 is in front of 3 other groups of students. Voting will be done by students. 12 groups will go to Round 2 and pitch in front of 3 jurors. 4 groups will move forward to the Finale Round in the Plenary where they will give the pitch to a group of jurors. There will be 1 winner group! They will receive seed capital of EU$500 and mentoring sessions to implement the project.
- Round 2 and 3 may include follow up questions (be ready to answer questions about the process, the project, the team, the target audience, the implementation and other topics! Round 1 doesn't include questions.

Suggested outline (not obligatory, you can get creative)
I. Introduction
II. Problem Statement
III. Solution Overview
IV. Design and Implementation
V. Impact and Sustainability
VI. Demo or Prototype (if applicable)
VII. Next Steps and Call to Action
VIII. Conclusion

Evaluation criteria - info for students

	Evaluation Criteria
Project	**Problem Solving**: Evaluate how well the project addresses the specific problem chosen.<br>The following elements should be present: The project is aligned and cohesive to the problem chosen, it makes sense and it tries to solve it. The project makes sense for that local community and the people in it.
	**Research Analysis**: Assess the analysis done by the team to understand the problem and the community<br>The following elements should be present: Students transmit the feeling that "they know what they're talking about", they show a good sense of understanding/knowledge of the problem and the community by using examples or cases. The project seems to be based on previous research and analysis of the topics either because they researched or they paid attention to the plenary sessions.
	**Innovation**: Assess the level of creativity and originality in the idea presented.<br>The following elements should be present: The project proposed is creative and disruptive, they offer a new perspective on how to address the problem in that community, they think of implementing the project in a way that is innovative.
	**Feasibility**: Consider the practicality and viability of the proposed solution.<br>The following elements should be present: The project can be implemented by the students in their local community, the project idea is attuned to the local context and it is possible to do.
	**Impact**: Evaluate the impact of the idea and how it will transform the community.<br>Think of the following questions: Does the project have the potential of being impactful? Can it create a long-lasting effect? Is the impact of the project high for that specific community?
	**Sustainability**: Assess the potential long-term impact and possible implementation/repeatance.<br>The following elements should be present: The project relevant for the long-term and it can be done multiple times in a sustainable way.
	**Scalability**: Consider the potential of the project to be implemented multiple times.<br>The following elements should be present: It's possible to scale the project to impact a growing number of people.
Pitch	**Communication & presentation:** Assess the clarity of the pitch, the ability of the team to articulate their idea, and their ability to engage and captivate the audience<br>The following elements should be present: The message is adjusted to the audience, the topic is explained with clarity, the pitch somehow reflects the team's own personality and the body language is appropriate. The pitch was within 5 min.
	**Team Dynamics**: Consider the composition and collaboration within the team.<br>The following elements should be present: The presentation flows, everybody knows when to speak, and when & how to intervene. The team seems in agreement with the project steps and they are supportive of each other.

Evaluation criteria for jurors

1 POINT	INSUFFICIENT	Evaluation Criteria	Name of the team			
2 POINTS	GOOD					
3 POINTS	VERY GOOD					
	Project	**Problem Solving**: Evaluate how well the project addresses the specific problem chosen.<br>The following elements should be present: The project is aligned and cohesive to the problem chosen, it makes sense and it tries to solve it. The project makes sense for that local community and the people in it.				
		**Research Analysis**: Assess the analysis done by the team to understand the problem and the community<br>The following elements should be present: Students transmit the feeling that "they know what they're talking about", they show a good sense of understanding/knowledge of the problem and the community by using examples or cases. The project seems to be based on previous research and analysis of the topics either because they researched or they paid attention to the plenary sessions.				
		**Innovation**: Assess the level of creativity and originality in the idea presented.<br>The following elements should be present: The project proposed is creative and disruptive, they offer a new perspective on how to address the problem in that community, they think of implementing the project in a way that is innovative.				
		**Feasibility**: Consider the practicality and viability of the proposed solution.<br>The following elements should be present: The project can be implemented by the students in their local community, the project idea is attuned to the local context and it is possible to do.				
		**Impact**: Evaluate the impact of the idea and how it will transform the community.<br>Think of the following questions: Does the project have the potential of being impactful? Can it create a long-lasting effect? Is the impact of the project high for that specific community?				
		**Sustainability**: Assess the potential long-term impact and possible implementation/repeatance.<br>The following elements should be present: The project relevant for the long-term and it can be done multiple times in a sustainable way.				
		**Scalability**: Consider the potential of the project to be implemented multiple times.<br>The following elements should be present: It's possible to scale the project to impact a growing number of people.				
	Pitch	**Communication & presentation:** Assess the clarity of the pitch, the ability of the team to articulate their idea, and their ability to engage and captivate the audience<br>The following elements should be present: The message is adjusted to the audience, the topic is explained with clarity, the pitch somehow reflects the team's own personality and the body language is appropriate. The pitch was within 5 min.				
		**Team Dynamics**: Consider the composition and collaboration within the team.<br>The following elements should be present: The presentation flows, everybody knows when to speak, and when & how to intervene. The team seems in agreement with the project steps and they are supportive of each other.				
		**Total**				
		**Comments**				

Follow up questions- info for jurors

		Follow up questions to ask the gorups in round 2 and 3			
	Design	E: Who and how are the target audiences of your project?		VH: Very hard question	
		E: What research or analysis did you conduct to further understand the specific problem you're addressing?		M: Moderate question	
		E: How is the context you have in mind for this project?		E: Easy question	
	Implementation	E: How do you plan to implement the project in your local community?			
		M: What main obstacles do you think you'll encounter when implementing it?			
		VH: How will you ensure adaptability as the project is implemented in various parts of the world?			
		M: What partnerships or collaborations do you envision to support the implementation and sustainability of your project?			
		M: How will you gather the team for implementing the project back home?			
		M: What resources or support do you need to implement and scale your project effectively?			
		M: What is your communication plan to create awareness and gather feedback?			
		E: How would you use money from a potential prize to implement your project?			
	Impact	M: How do you think the people/beneficiaries will benefit from this initiative?			
		VH: How do you measure the success and impact of your project? What metrics or indicators will you use?			
		M: How will you engage and involve the community in the project to ensure sustained impact?			
	Pitch development Process	E: What highlights can you mention from the process these days?			
		E: From 1-5, How was it working together?			
		E: What main obstacles have you faced these days?			
		E: How do you feel about your own project?			
