# Supplementary material for: Co-Creation of Mental Health Intervention for Adolescents: A Social Hackathon Approach
Source: Healthcare (Basel). 2026 May 12;14(10):1315. doi: 10.3390/healthcare14101315 (PMC13205654; doi:10.3390/healthcare14101315)
Supplement: Supplementary file 1 [file healthcare-14-01315-s001.zip › 3. Project transcripts.pdf]

Transcripts of Project Outlines

Pink Duck B1

Please briefly describe your project idea:
We plan to organize community events to encourage student socialization and connect them with peers who share similar hobbies. The project includes inviting professionals to schools to discuss mental health and organizing events to combat loneliness. We also aim to provide professional assistance for existing problems.

What is the aim of your project?
To build a supportive community for students, helping them overcome feelings of loneliness and fostering a sense of belonging.

How do you plan to implement your idea?
By surveying students to identify their preferred hobby groups and the types of professionals they would like to hear from. This approach ensures we are catering to their interests and needs.

What will you need to implement your project? (Infrastructure? People? Support?)
We will need organizational support and people interested in participating in these events.

Do you need money to implement your project? How much?
Yes, funding will be required to invite professionals.

What obstacles could you encounter?
Securing the necessary funding to invite professionals could be a challenge.

Pink Elephant C1

Please briefly describe your project idea:
Our project is centered on organizing community events to enhance student socialization and help them connect with peers who have similar interests. The initiative includes inviting professionals to schools to speak on mental health issues and arranging events to combat loneliness. Additionally, we plan to provide professional assistance for those dealing with existing challenges.

What is the aim of your project?

The main aim of our project is to create a supportive community environment for students, helping them to overcome feelings of loneliness and fostering a sense of belonging among them.

How do you plan to implement your idea?

We plan to implement our idea by conducting surveys to identify the hobby groups students are interested in and the types of professionals they would like to interact with. This approach will help ensure that our activities are relevant and engaging for the students.

What will you need to implement your project? (Infrastructure? People? Support?)

To implement our project effectively, we will require organizational support and the involvement of people who are interested in participating in and contributing to these events.

Do you need money to implement your project? How much?

Yes, we will require financial resources to hire professionals and cover the costs of organizing the events. The total amount needed will depend on the scale of the events and the fees of the professionals involved.

What obstacles could you encounter?

A major challenge we anticipate is securing enough funding to pay for the professional speakers and to cover the logistical costs of the events.

Pink Fox C1

Please briefly describe your project idea:
The project aims to make learning fun and easy, creating an awareness of different types of education and promoting mental strength so that students do not feel afraid.

What is the aim of your project?
The aim of the project is to reduce stress experienced by students at school.

How do you plan to implement your idea:
Implement by promoting communication through therapies, open workshops, and other strategies.

What will you need to implement your project? (Infrastructure? People? Support?)
Therapists, educators, and support are needed for implementation.

Do you need money to implement your project? How much?
Yes, payment of €500 or more is required.

What obstacles could you encounter?
Potential obstacles include resistance from a conservative education system that may not easily accept change or progress, particularly from teachers and the education sector.

Pink goat C3 stress and anxiety

Please briefly describe your project idea:
Our project aims to introduce a campaign to support education about anxiety and stress. The campaign will consist of educational initiatives in schools, online lessons, posters, a website, and soundproof rooms equipped with letterboxes for students to release and write down their stress.

What is the aim of your project?
The group aims to reduce stress and anxiety in schools and encourage people to speak up about their stress and anxiety.

How do you plan to implement your idea:

Propose ideas and present the campaign to schools.
Create online lessons.
Develop a website.
Establish soundproof rooms in schools.

What will you need to implement your project? (Infrastructure? People? Support?)

- Schools as partners
- Website development
- Professional for online lessons

Do you need money to implement your project? How much?

- Salary for the professional (online lessons): €1,000
- Soundproof room in the school

What obstacles could you encounter?

- Difficulty finding schools willing to participate
- Agreement on the project, especially in France
- Motivation of certain individuals
- Finding people willing to work on the project

Pink Horse A3

Please briefly describe your project idea:

Creating a new YFU branch that spreads self-love, confidence, and self-worth, specifically geared towards the younger generations. We would hold speeches and workshops at schools, discussing self-love with the help of volunteers.

What is the aim of your project?

The aim of the project is to positively change the way people think about themselves.

How do you plan to implement your idea:

Apart from speeches and workshops, we would create social media accounts where we will post weekly affirmations and self-love assignments for viewers. We will also upload videos and podcasts and distribute stickers while hanging up posters with motivational messages.

What will you need to implement your project? (Infrastructure? People? Support?)

We would need support from YFU and YFU volunteers and funding for marketing.

Do you need money to implement your project? How much?

We would need money for marketing, such as posters and stickers (up to 500€).

What obstacles could you encounter?

Possible obstacles include not having enough volunteers and support.

Pink Iguana A1 social media

Please briefly describe your project idea:
We propose a workshop in schools to educate students on navigating social media in a healthy manner.

What is the aim of your project?
The aim is to raise awareness about the potential dangers of social media.

How do you plan to implement your idea:
We plan to conduct workshops that will replace a dedicated lesson in each class timetable, ensuring that each student is exposed to and engages with the material.

What will you need to implement your project? (Infrastructure? People? Support?)
We will require volunteers, professionals, posters, and support for the implementation.

Do you need money to implement your project? How much?
We estimate a budget of €950 for the planned activities.

What obstacles could you encounter?
Potential obstacles include difficulty in finding professionals or volunteers and challenges in fundraising not meeting the intended goals.

Purple Albatross B3

Please briefly describe your project idea:

Our project focuses on raising awareness about the challenges neurodivergent individuals face in school settings. We plan to inform about the accommodations schools can provide to support these students. Our approach includes social media campaigns, incorporating statistics about neurodiversity, and highlighting communities affected by these issues. We plan to use QR codes for easy information access.

What is the aim of your project?

The aim is to spread awareness and educate people about neurodiversity and the importance of accommodating neurodivergent students in educational settings.

How do you plan to implement your idea?

- Distribution of QR codes.
- Informational meetings.
- Creating and displaying posters.
- Involving student councils.

What will you need to implement your project? (Infrastructure? People? Support?)

- A provider for QR code generation.
- Stationery supplies (pens, paper for posters).
- Support from educational institutions.

Do you need money to implement your project? How much?

[Details about the budget required are not specified.]

What obstacles could you encounter?

- Low interest in the social media campaign.
- Competition from existing campaigns and information sources.

Purple Bee B3 value and diversity

Please briefly describe your project idea:
Our project aims to conduct workshops at schools with the assistance of professionals experienced in the subject of neurodivergent individuals and those with disabilities through simulations.

What is the aim of your project?
To help and raise awareness about disabilities in schools and lessen prejudice.

How do you plan to implement your idea:

Engage professionals experienced in the field to conduct workshops for students, teachers, and staff.
Collaborate with the school to establish a new community.
Consult with a programmer to develop simulations.

What will you need to implement your project? (Infrastructure? People? Support?)

- Room
- Experts, teachers, and students
- Computers, paper/pens, questions and exercises, music boxes, microphones, projector

Do you need money to implement your project? How much?
Yes, we will try to find volunteers, but if not, we need funding for materials, professionals, school equipment (beamer/laptops).

What obstacles could you encounter?

- Lack of motivation from students.
- Difficulty finding a suitable day to present the project.

Purple Cat C1

Please briefly describe your project idea:

Our project is a workshop (spanning 2-3 days) focused on teaching students effective stress management and study techniques. The workshop will include sessions led by experts aimed at educating students on how to cope with academic pressure.

What is the aim of your project?

The aim is to reduce stress among students by fostering awareness, empathy, and a more comfortable learning atmosphere.

How do you plan to implement your idea?

We plan to collaborate with schools to organize these workshops, ensuring participation from all parties involved in the educational process.

What will you need to implement your project? (Infrastructure? People? Support?)

We will need workshop leaders (professionals in stress management and education), as well as volunteers to assist in the background.

Do you need money to implement your project? How much?

Yes, funding is required for the professionals' salaries and for materials such as paper and markers.

What obstacles could you encounter?

Potential challenges include lack of participation from students, parents, or teachers, and general disinterest in the program.

Purple Duck B3

Please briefly describe your project idea:

Our goal is to establish an organisation dedicated to raising awareness about neurodivergent individuals and their struggles. We aim to use various methods, including social media, to disseminate information.

What is the aim of your project?

We aim to increase public awareness of neurodivergent conditions through social media and to collaborate with volunteers passionate about this cause.

How do you plan to implement your idea?

- Utilising social media platforms.
- Conducting workshops.
- Distributing flyers and posters in schools.

What will you need to implement your project? (Infrastructure? People? Support?)

- Professional support.
- Volunteers.
- Financial backing.
- A venue for events and speeches.

Do you need money to implement your project? How much?

- Advertising: €100
- Travel expenses: €100
- Accommodation: €50
- Equipment: €500
- Unforeseen expenses: €250
- Total: €1000

What obstacles could you encounter?

- Financial constraints.
- Lack of interest from potential participants.

Purple Elephant C1

Please briefly describe your project idea:
Our project is to redesign the architectural structure of a typical school to create more open and comfortable spaces. We believe that providing students with a free and relaxed environment will enhance their focus and learning experience.

What is the aim of your project?
The aim is to make learning environments more comfortable for students, thereby reducing school-induced stress.

How do you plan to implement your idea?
We plan to start with remodelling one classroom in a model school. We'll observe if this change increases student concentration levels, and based on the results, we would consider remodelling the entire school building.

What will you need to implement your project? (Infrastructure? People? Support?)
We need the support of students, parents, and the school board. For the practical aspect, we require a few workers for the structural changes and an interior designer to redesign the classrooms in a functional yet enjoyable manner.

Do you need money to implement your project? How much?
For the initial test classroom, our estimated budget is around $500. This amount should be sufficient for the initial phase.

What obstacles could you encounter?
A potential challenge is resistance from some parents, but we hope to overcome this through discussions and showing the positive impacts of the changes.

Purple Fox C1

Please briefly describe your project idea:
Our project proposes a fundamental change in school practices to create a lasting solution to the problem of student stress.

What is the aim of your project?
The aim is to reform school rules and practices to better support students, thereby reducing their stress and anxiety.

How do you plan to implement your idea?
We plan to pitch our idea to the school staff, using examples from other countries' school systems to support our proposal.

What will you need to implement your project? (Infrastructure? People? Support?)
We will need posters for awareness and public speakers (motivational staff) to engage and inspire the school community.

Do you need money to implement your project? How much?
Yes, we estimate a budget of €100 for posters and approximately €190 for public speaking and presentation resources.

What obstacles could you encounter?
Potential challenges include reluctance or resistance from school staff or leadership, and apathy or lack of engagement from students.

Purple Goats B1

Please briefly describe your project idea:
An app that can help lonely exchange students in Germany find other exchange students close to their location. It's an app where you can connect with other exchange students, share experiences, and build a supportive community.

What is the aim of your project?
The aim of the app is to enhance the exchange experience from the beginning, overcoming loneliness, and fostering mutual support among students.

How do you plan to implement your idea?

Identify a skilled programmer for app development.
Brainstorm additional features for the app.
Gather feedback from other exchange students.

What will you need to implement your project? (Infrastructure? People? Support?)
Volunteer programmers.

Do you need money to implement your project? How much?
Yes, we will need funding to launch the app. The specific amount will be determined after consulting with the programmer.

What obstacles could you encounter?

- App development costs.
- Difficulty in finding a suitable programmer.

Purple Horse A1

Please briefly describe your project idea:
Love Notes 75 of social media thinking and group, a project that tackles the negative impact teenagers face by promoting self-love and critical open dialogue through workshops and personal journaling discussions.

What is the aim of your project?
The aim of the project is to address the negative impact of social media on the self-esteem and well-being of teenagers, fostering self-love, critical thinking, and empowering teenagers to have a healthier relationship with the digital world.

How do you plan to implement your idea:
The implementation of Love Notes involves the following steps:

- Providing journals to students where they can write daily love notes
- Developing discussilets, a workshop format that combines individual journaling and group discussions, creating a safe and supportive environment.

What will you need to implement your project? (Infrastructure? People? Support?)

- Support from schools
- Volunteers
- Youth

Do you need money to implement your project? How much?
The budget given for the Hackathon (500€) will be enough to make the project happen.

What obstacles could you encounter?

- Not finding enough volunteers
- Not getting permission/support from schools
- Lack of interest from teenagers

Black albatross C3 stress and anxiety

Please briefly describe your project idea:

Cozy Cave: Create a safe space at school (approximately 200m) where students can come to relieve stress and unwind (quiet and simple).

Council Sessions: Every two weeks, students will gather in groups of eight to share their feelings and struggles.

What is the aim of your project?
To reduce the stigma around stress and anxiety and provide students with a safe space at school.

How do you plan to implement your idea:
We will ask volunteers, including local university psychology students, to lead the sessions. These sessions will take place after school hours every two weeks and will be treated as general support (attendance is mandatory). The room will be arranged at minimal cost.

What will you need to implement your project? (Infrastructure? People? Support?)
We need volunteers, including university psychology students, and a room.

Do you need money to implement your project? How much?
We would need money for the furniture of the room (+500).

What obstacles could you encounter?
People may skip the sessions, and the room may be mistreated.

Black Elephant C3

Please briefly describe your project idea:
We plan to create a "Wellness Box," where students can anonymously leave positive notes for those in need of encouragement, maintaining their privacy and comfort.

What is the aim of your project?
The aim is to foster a positive atmosphere within the school by spreading encouraging and supportive messages.

How do you plan to implement your idea?
We intend to conduct a creative workshop to introduce and explain the concept to everyone.

What will you need to implement your project? (Infrastructure? People? Support?)
Our project heavily relies on student engagement and the success of our workshop. We will need a physical box and creative materials for the workshop.

Do you need money to implement your project? How much?
We will need funding for constructing the box and enhancing the workshop's appeal.

What obstacles could you encounter?
Possible challenges include the box being vandalised or misused for placing negative or harmful notes.

Black Fox B1 Loneliness

Please briefly describe your project idea:
Our project aims to create stickers that bring people with similar interests together, spreading awareness about togetherness. We will advertise these stickers in different communities, such as schools and neighborhoods, to promote social events and foster a sense of loveliness.

What is the aim of your project?
The goal is to bring people together and reduce loneliness.

How do you plan to implement your idea:
We plan to distribute stickers in various community settings, including schools and neighborhoods, to encourage social activities and promote loveliness. Additionally, we will create social events and spread information through an Instagram account.

What will you need to implement your project? (Infrastructure? People? Support?)
We require the support of communities, a printer for sticker production, funding for advertising, assistance with event organization, and individuals to manage the Instagram account.

Do you need money to implement your project? How much?
Yes, we estimate a budget of 500€ for printing, advertising, and organizing events.

What obstacles could you encounter?
Potential challenges include difficulty obtaining support from communities and insufficient funding for widespread sticker distribution.

Black goat C1

Please briefly describe your project idea:
The project aims to connect high school students and university students who have previously experienced similar causes of stress. The conversation will cover daily and systematic school problems. The project committee, consisting of student ambassadors, teachers, university students, and a mental health expert, will oversee this initiative.

What is the aim of your project?
To create a socially stable environment that prevents serious stress dangers and to establish a lasting culture of mental support.

How do you plan to implement your idea:
To implement our idea, we need to build a committee led by a mental health expert. With professional assistance, we will create a survey to identify the personal stress points. This information will be used in matching students.

What will you need to implement your project? (Infrastructure? People? Support?)
We will need volunteers from schools and universities, along with financial support to compensate committee members on a monthly basis.

Do you need money to implement your project? How much?
Yes, for the pilot project over 10 months, we would need approximately 7000€.

What obstacles could you encounter?
Possible obstacles include a shortage of university student volunteers in the initial phase of the project. However, this issue can be resolved over the long term as students who have previously received support in high school are more likely to volunteer as university students.

Black Horses C3

Please briefly describe your project idea:
SELF-CARE APP: A mobile application where students can relax, hear affirmations, and talk anonymously about their problems.

What is the aim of your project?
To reduce stress

How do you plan to implement your idea:
create an app

What will you need to implement your project? (Infrastructure? People? Support?)
PROGRAMMERS, PSYCHOLOGISTS, MONEY, TEAM, GRAPHIC DESIGNER.

Do you need money to implement your project? How much?
Approximately 2800€.

What obstacles could you encounter?
Programming difficulties, recruitment issues, and challenges in advertising.

Black Iguana B1

Please briefly describe your project idea:
We propose an app where high-school students with similar interests can connect and meet. The aim is to provide a platform for lonely or isolated individuals to easily socialize with others.

What is the aim of your project?
The aim is to facilitate socialization for lonely/isolated individuals.

How do you plan to implement your idea:
We will develop the app and begin with advertising and testing. If everything goes well, we may expand to a professional website or program with the help of a professional programmer.

What will you need to implement your project? (Infrastructure? People? Support?)
We will need:

- Professional programmers -> money to pay their wages
- Volunteer counselors
- Advertising support

Do you need money to implement your project? How much?
We would need $500, but preferably enough for programming as well.

What obstacles could you encounter?
Possible obstacles include not having enough money to pay for professional programmers and counselors. Additionally, finding schools or organizations willing to support or volunteer may pose a challenge.

Blue albatros A3

Please briefly describe your project idea:
Create a website where users can inform themselves about improving self-worth, confidence, and self-love. The platform will offer guided meditations and teach breathing control. Users can seek free professional help, share their experiences, and sign up for free seminars held by volunteers. Every day, we aim to create a challenge to immerse the user in a positive mindset. For example, you might need to write about positive things about yourself.

What is the aim of your project?
Our aim is to assist as many young individuals as possible on their journey to self-love, confidence, and self-worth. We also want to provide students with the opportunity to access free professional mental health support.

How do you plan to implement your idea:
We plan to start testing the website with users, gather feedback, make necessary improvements, and then advertise it to reach a wider audience.

What will you need to implement your project? (Infrastructure? People? Support?)
To realize our project, we need volunteers willing to share their knowledge and assistance from a web designer.

Do you need money to implement your project? How much?
We only need passionate volunteers to help bring our project to life. However, we would appreciate around €200 for providing a professional programmer.

What obstacles could you encounter?
One potential obstacle is gaining recognition for our website, which can be addressed by sharing through social media and word of mouth.

Blue Bees B3

Please briefly describe your project idea:
We plan to conduct workshops where individuals with various disabilities will engage middle school students, teaching them about life with a disability. This aims to involve students actively in their own learning and to foster an inclusive society.

What is the aim of your project?
To raise awareness about the challenges associated with disabilities and promote a fair and inclusive society.

How do you plan to implement your idea?
By organising workshops every three months with new speakers throughout the school year.

What will you need to implement your project? (Infrastructure? People? Support?)
We will need volunteer speakers to educate the students.

Do you need money to implement your project? How much?
We may need funds for volunteer accommodations, if required.

What obstacles could you encounter?
Finding volunteers and schools willing to host our workshops could be challenging.

Blue Cat C2

Please briefly describe your project idea:
Our project is centred on educating students and teachers, with a focus on private sessions to monitor and handle bullying cases effectively, thus ensuring the well-being of students.

What is the aim of your project?
The aim is to assist students who are bullied, offer support, and work towards reducing the incidence of bullying in schools.

How do you plan to implement your idea?
We plan to have volunteer teachers receive training from professionals before the school year begins. Once the school year starts, these trained teachers will be available to assist any student needing help. A team of volunteer teachers and a psychologist will be formed initially.

What will you need to implement your project? (Infrastructure? People? Support?)
We will require the expertise of a psychologist to train the teachers.

Do you need money to implement your project? How much?
[Details about the budget required are not specified.]

What obstacles could you encounter?
Potential challenges may include finding willing volunteer teachers and psychologists, and ensuring effective implementation and monitoring of the program.

Blue ducks A1 social media

Please briefly describe your project idea:
We plan to conduct a workshop for YFUVILLE high school students about the effects of social media on body image, incorporating a surprising twist at the end.

What is the aim of your project?
To create a noticeable contrast between the workshop and the accompanying video.

How do you plan to implement your idea:
As a group, we will conduct an experiment demonstrating how editing can influence people's mindset regarding social media and body image. This will involve visiting YFUVILLE high school.

What will you need to implement your project? (Infrastructure? People? Support?)
We require a cameraman and an editor. The group members are volunteers for the experiment.

Do you need money to implement your project? How much?
Yes, we need some funds, depending on the country's salary rates. For Germany, approximately 17 Euros per hour for the cameraman and 85 Euros per hour for the editor.

What obstacles could you encounter?
One potential challenge is ensuring that the workshop and video exhibit a significant contrast, recognizing the inherent risk of working with human reactions.

Blue elephant B1 loneliness

Please briefly describe your project idea:
The idea is to address loneliness among teens and seniors. The program involves teens visiting seniors at a nursing home, taking field trips, and engaging in activities like visiting museums and cafes throughout the year.

What is the aim of your project?
The aim is to help lonely teens and seniors find friends, encourage conversations between different generations, and foster new friendships.

How do you plan to implement your idea:
We need support from a nursing home and a school to execute our program.

What will you need to implement your project? (Infrastructure? People? Support?)

- Partnership with a nursing home
- School collaboration for program implementation
- Funding for field trips and program materials

Do you need money to implement your project? How much?
We need funds for the field trips that teens and seniors will participate in, twice a week, and for organizing a concluding event between the seniors and teens.

What obstacles could you encounter?
The main obstacle could be a lack of interest from either the seniors or teens in our program.

Blue Fox A3 self-worth, confidence, self -love

Please briefly describe your project idea:
The project involves creating a website to promote self-love, self-confidence, and sharing tips, videos, and practices to help and support young people facing self-judgment. Social media platforms will be utilized with the help of influencers and social media communities.

What is the aim of your project?
To make young people realize that they are not alone in their situation and that the idealized "pop" they see on social media also faces self-judgment problems.

How do you plan to implement your idea:
Create a website and social media accounts to help young people. Use ads in collaboration with influencers and create content.

What will you need to implement your project? (Infrastructure? People? Support?)

- Influencers
- Funding for the website and ads
- Professionals knowledgeable about the topic

Do you need money to implement your project? How much?
As much as possible for ads, but only necessary funds for creating and maintaining the website.

What obstacles could you encounter?
The main obstacle could be not reaching a sufficient audience.

Blue Goat A2

Please briefly describe your project idea:
Our idea is to develop an app to enhance the overall health and well-being of high school students. Unique features include daily affirmations termed "casual magic," personalised positive messages, a community events calendar for inclusion, and a journal feature.

What is the aim of your project?
The aim is to help students recognize their self-worth and understand that their inner qualities define them, thereby improving their physical and mental health.

How do you plan to implement your idea?
Initially, we will introduce the app in a specific high school, using social media and the support of school staff like teachers. We also plan to conduct a study to assess the project's impact.

What will you need to implement your project? (Infrastructure? People? Support?)
We will require technical support for app development and teachers to promote it.

Do you need money to implement your project? How much?
As we have limited coding experience, we will need financial support for professional coding assistance, though the exact amount is currently unknown.

What obstacles could you encounter?
We may face technical challenges and difficulties in attracting users.

Blue horse C1

Please briefly describe your project idea:
Create a website/app where students who perform poorly on a test can earn a small amount of bonus points to add to their grade by sharing their notes with other students. The platform will include exercises for specific sections. Additionally, it will provide information videos about mental health, focusing on the toll of excessive pressure and stress on a student's well-being.

What is the aim of your project?
To reduce the stress of students who are missing notes or struggling with mental health issues and inform them about dealing with mental health struggles.

How do you plan to implement your idea:
The website/app will be introduced to the school where the organizers attend for a test round. Results will be tracked, and an improved version will be introduced to other schools based on these results.

What will you need to implement your project? (Infrastructure? People? Support?)

- A professional-looking website with the help of a webdesigner / programmer
- Testers who are willing to try the beta version
- Advertisements (on social media, teacher forums, flyers in schools)
- Time

Do you need money to implement your project? How much?
€500 - €1000

What obstacles could you encounter?

- The algorithm creation being too expensive
- Lack of interest from schools

Blue Iguana B2

Please briefly describe your project idea:
To decrease the bullying percentage within schools, we aim to create an app that allows students to report problems to the school council anonymously.

What is the aim of your project?
The aim is to make bullied students feel less afraid of reporting bullying and provide them with the opportunity to anonymously report incidents without fear.

How do you plan to implement your idea:
The first step is to design the app and perform the coding. We will carefully choose all the functions and conduct a trial for a group.

What will you need to implement your project? (Infrastructure? People? Support?)
Support to build the app or, optionally, a website and help to get it utilized in schools.

Do you need money to implement your project? How much?
We couldn't finalize the budget yet because we need to determine the cost of coding, but it will probably exceed the budget.

What obstacles could you encounter?
Potential obstacles include the coding of the app, presenting it to all schools, and establishing reliability in their eyes.

Orange albatros B2 Bullying

Please briefly describe your project idea:
Our project, "Don't Hate, Congratulate," aims to spread awareness about the growing problem of bullying. We believe that bullying has a significant impact on individuals, making it a major issue. To address this, we want to start a campaign to raise awareness and prevent bullying. The goal is to make bullying less prevalent by encouraging more people to stand up against it.

What is the aim of your project?
The aim of our project is to raise awareness about bullying, educate teachers and students on how to prevent it, and create a positive school environment where everyone can work together without fear of bullying. We also plan to reward the "best friend of the month" as an incentive for promoting positive behavior.

How do you plan to implement your idea:
To implement our idea, we need to educate teachers and students about the impact of bullying and how to prevent it. We will work with school counselors to provide support and create a safe space for students to discuss any problems or experiences anonymously. Additionally, we will organize school events and challenges to engage students positively.

What will you need to implement your project? (Infrastructure? People? Support?)
We need support to educate teachers and students, collaboration with school counselors, and financial assistance for organizing events and challenges.

Do you need money to implement your project? How much?
Yes, we need funds for educating teachers, working with counselors, and organizing events. The exact amount required depends on the scope of the campaign.

What obstacles could you encounter?
Possible obstacles include a lack of student interest, insufficient funds, the effectiveness of the program, and low attendance at events.

Orange Bee C2

Please briefly describe your project idea:
Our initiative focuses on mentoring and educating students about the unrealistic standards of beauty and societal pressures prevalent in schools. We aim to steer students towards adopting healthier lifestyles and self-perceptions. A key component of our project is the creation of a social media platform, designed to facilitate the sharing of personal experiences and to maintain ongoing engagement with students post-sessions.

What is the aim of your project?
The primary objective is to shed light on the detrimental effects of unrealistic beauty standards on self-esteem and the societal pressures to conform. Our goal is to empower students to appreciate their own worth and to challenge societal expectations.

How do you plan to implement your idea?
We intend to conduct visits to various schools to initiate discussions on societal ideals related to appearance. Additionally, we plan to launch a social media campaign that encourages students to share their personal stories. This campaign is aimed at providing both mental and physical empowerment to students.

What will you need to implement your project? (Infrastructure? People? Support?)
For effective implementation, we require a projector and access to room space within schools. We also need the expertise of a graphic designer to create educational posters and flyers, along with the necessary materials for production.

Do you need money to implement your project? How much?
The financial requirements will depend on the extent of the project's reach and the number of schools we engage with.

What obstacles could you encounter?
Potential challenges may include securing permissions to access schools, overcoming language barriers in diverse locations, and addressing logistical challenges, particularly in transportation.

Orange Cat C2

Please briefly describe your project idea:
Our project focuses on organising community events to encourage student socialisation and foster connections with peers who share similar interests. The project involves inviting professionals to schools to address mental health issues and hosting events aimed at reducing loneliness. We also plan to offer professional support for those facing existing personal challenges.

What is the aim of your project?
The primary goal of our project is to establish a nurturing community environment for students, aiding them in overcoming loneliness and promoting a sense of belonging.

How do you plan to implement your idea?
We intend to implement our idea by surveying students to ascertain their interests in various hobby groups and the types of professionals they would like to meet. This strategy will help tailor our activities to be both relevant and appealing to the students.

What will you need to implement your project? (Infrastructure? People? Support?)
For effective implementation, our project will require organisational support and active participation from individuals who are interested in attending and contributing to the events.

Do you need money to implement your project? How much?
Yes, funding is necessary to hire professionals and manage the logistics of organising the events. The total budget will depend on the scope of the events and the professional fees involved.

What obstacles could you encounter?
One significant challenge we foresee is obtaining sufficient funding to cover the costs of professional speakers and the overall organisation of the events.

Orange Duck B1

Please briefly describe your project idea:
Our initiative is dedicated to organizing community events aimed at enhancing student socialization and helping them connect with peers who have shared interests. Key aspects of the project include inviting professionals to schools for mental health discussions and organizing activities to combat loneliness. Additionally, our plan includes providing professional support for students facing existing personal challenges.

What is the aim of your project?
The main objective of our project is to create a supportive and nurturing community environment for students. This aims to assist them in overcoming loneliness and cultivating a sense of belonging.

How do you plan to implement your idea?
Our plan for implementation involves surveying students to identify their interests in specific hobby groups and determining the types of professionals they would be interested in meeting. This approach is designed to ensure that our activities are both relevant and engaging for the student body.

What will you need to implement your project? (Infrastructure? People? Support?)
To successfully carry out our project, we will need organizational support and the involvement of people who are keen to participate in and contribute to these events.

Do you need money to implement your project? How much?
Yes, financial resources are essential for hiring professionals and handling the logistics of organizing these events. The total amount required will depend on the scale of the events and the fees for the professionals involved.

What obstacles could you encounter?
One of the major challenges we anticipate is securing adequate funding to cover the expenses of professional speakers and the overall organization of the events.

Orange Elephant A2

Please briefly describe your project idea:
Our project involves mentoring and raising awareness about unrealistic beauty standards and societal pressures in schools. We aim to guide students towards healthier lifestyles and self-perceptions. Additionally, we plan to develop a social media platform to encourage sharing of experiences and to maintain contact with students after the sessions.

What is the aim of your project?
The aim is to highlight how unrealistic beauty standards negatively impact self-esteem and create societal pressure to conform. We intend to empower students to recognize and value their own worth beyond societal expectations.

How do you plan to implement your idea?
We plan to visit various schools to discuss societal ideals related to appearance and launch a social media campaign encouraging students to share their stories. This campaign aims to educate and empower students mentally and physically.

What will you need to implement your project? (Infrastructure? People? Support?)
We will need a projector, room space in schools, and a graphic designer for creating educational posters and flyers. Materials for producing these items are also necessary.

Do you need money to implement your project? How much?
The costs will vary depending on the project's scope and the number of schools involved.

What obstacles could you encounter?
Challenges may include obtaining permission to enter schools, language barriers in different locations, and logistical issues related to transportation.

Orange Foxes C1

Please briefly describe your project idea:
Our project involves organising community events aimed at enhancing student socialisation and connecting them with others who have similar interests. The initiative includes inviting professionals to schools to talk about mental health and hosting events to help alleviate loneliness. Additionally, we plan to offer professional support to address existing issues.

What is the aim of your project?
The goal is to create a supportive community for students, assisting them in overcoming loneliness and cultivating a sense of belonging.
How do you plan to implement your idea?

We intend to implement our idea by conducting surveys among students to identify their preferred hobby groups and determine the types of professionals they would be interested in hearing from. This method ensures that our activities align with their interests and needs.

What will you need to implement your project? (Infrastructure? People? Support?)
To implement our project, we require organisational support and engagement from individuals interested in participating in our events.

Do you need money to implement your project? How much?
Yes, we will need funding to cover the costs of inviting professionals.

What obstacles could you encounter?
One of the main challenges we anticipate is securing sufficient funding to afford the professional speakers and facilitators for our events.

Orange Goat B1

Please briefly describe your project idea:
Our initiative is focused on organizing community events to foster student socialization and connect them with peers who share similar hobbies. The project entails inviting professionals to schools to discuss mental health, along with arranging events designed to reduce loneliness. We also aim to provide professional help for those facing existing challenges.

What is the aim of your project?
The primary objective of our project is to establish a supportive community for students, aiding them in overcoming feelings of loneliness and promoting a sense of belonging.

How do you plan to implement your idea?
Our implementation strategy involves surveying students to identify their preferred hobby groups and the types of professionals they are interested in engaging with. This approach ensures that our activities are tailored to their interests and needs.

What will you need to implement your project? (Infrastructure? People? Support?)
To successfully execute our project, we need organisational support, as well as active participation from individuals interested in our events.

Do you need money to implement your project? How much?
Yes, financial resources are necessary to compensate the professionals we plan to invite. The exact amount of funding required will be determined based on the scale of the events and the professionals involved.

What obstacles could you encounter?
One significant challenge we foresee is obtaining adequate funding to cover the costs associated with hiring professional speakers and organising the events.

Orange Horse B2

Please briefly describe your project idea:
Our project aims to elevate the National Anti-Bullying Day to an international level, promoting global awareness of bullying. We plan to propose solutions and strategies to combat bullying effectively.

What is the aim of your project?
The aim is to raise awareness about bullying, highlighting National Anti-Bullying Day, and to propose practical solutions for its prevention.

How do you plan to implement your idea?
We intend to collaborate with school administrators and local anti-bullying organizations to bring this initiative to fruition. This involves organizing awareness campaigns and workshops in schools.

What will you need to implement your project? (Infrastructure? People? Support?)
Support from schools is crucial. We plan to contact local anti-bullying organizations to assist in educating teachers and students about bullying.

Do you need money to implement your project? How much?
We aim to raise funds for the anti-bullying organization to support our initiative. The exact amount needed will be determined at a later stage.

What obstacles could you encounter?
Challenges may include getting schools and professionals on board with the project.

Orange Iguana A1

Please briefly describe your project idea:

We plan to develop a workshop program that schools can use to address social media-related issues. The workshop will focus on educating students about the responsible use of social media.

What is the aim of your project?

The aim is to educate students on better and safer social media usage, promoting responsible online behavior.

How do you plan to implement your idea?

We intend to create an easy-to-use workshop program for schools, designed to combat problems arising from social media usage.

What will you need to implement your project? (Infrastructure? People? Support?)

We will need resources to develop the program, including a script and tools that schools can use effectively.

Do you need money to implement your project? How much?

Yes, we estimate the budget to be around €5,000.

What obstacles could you encounter?

One of the main challenges could be schools not adopting or being hesitant to implement the program.

Pink Albatros A2

Please briefly describe your project idea:
Create a mirror with a curtain that can be pulled down to prevent people from looking at themselves for too long. Many problems with self-esteem and body image stem from overanalyzing features, and the mirror with a curtain would address that issue.

What is the aim of your project?
To reduce the time that people spend looking in the mirror and encourage practical use of the mirror for its intended purposes.

How do you plan to implement your idea:
Screw the curtain on top of the mirror that can be rolled down.

What will you need to implement your project? (Infrastructure? People? Support?)
We will need support and people to inform about the idea (marketing).

Do you need money to implement your project? How much?
Yes, it depends on how many mirrors we choose to make.

What obstacles could you encounter?
Possible obstacles include the mirror not being bought and potential misuse by people.

Pink Bee A2

Please briefly describe your project idea:
We have planned to create a project with the purpose of educating the students of YFUVILLE about ways to overcome harmful societal ideas and beauty standards. This project will be an event that implements multi-faceted approaches to tackle these issues, including physical activity, nutrition, self-care, and evaluation. Our project aims to create a supportive environment through various angles such as education and fun activities.

What is the aim of your project?
The project is all about embracing oneself, promoting mental, and emotional well-being, from appearance to overall health. We hope to shift the focus at YFUVILLE high school, thereby promoting a positive and healthy environment.

How do you plan to implement your idea:
We plan to lead a three-day event with various activities that serve the purpose of spreading awareness about the negative impact of harmful societal ideas and beauty standards.

What will you need to implement your project? (Infrastructure? People? Support?)
We will need the school site for the event (kitchen, classrooms, outside area, auditorium, etc.), people to lead sessions and talk (psychologists, volunteers, special guests, cooks), and funding.

Do you need money to implement your project? How much?
Approximately 500-600 €.

What obstacles could you encounter?
Possible obstacles include a lack of available people for activities (speakers, cooks), funding issues, unwillingness to participate, and a lack of infrastructure.

Pink Cat C1

Please briefly describe your project idea:
Our project aims to set limits to reduce workload. We plan to invite someone with experience in handling stress to give a presentation at the school. Following that, we will conduct a survey to gather information about the student workload. With this information, we aim to establish limits to keep stress neutral and not overwhelming.

What is the aim of your project?
The aim is to establish limits to keep stress neutral and not overwhelming.

How do you plan to implement your idea:

- Form a team of students
- Organize the expert for the presentation
- Create the survey and track data

What will you need to implement your project? (Infrastructure? People? Support?)

- Stress expert for the presentation
- Survey

Do you need money to implement your project? How much?
Approximately 100-200€ (depends) for the expert.

What obstacles could you encounter?

- Students trolling the survey
- Students not paying attention during the presentation

Transcripts of audio recordings

BLACK DUCK (A1)

S1: Route one black duck.

S2: Yeah. So your time will officially start now.

UU: So. Hello. Welcome to our.

S2: Presentation. Uh, we are from Black Box Group, and our project is crack in mind. Uh, the problem that we choose is about, um, how might we empower the youth to cultivate, uh, a healthier relationship with social media? Let's get started.

S3: Okay, so our problem is that too many students are badly influenced by social media due to a lack of how it actually works. So there will be times that you feel good and included in everything. And there will also be times that you feel like not being good enough that will lead to consequences, which can be, um, starting with building up an insecurity or. All the perfect body images you see on the internet can lead to mental health issues like having anxiety or something. To be scared to show yourself to others. Um, and it will lead you to a kind of obsession with social media, like having the urge to look like all of the perfect bodies that you see on the internet. After all, you can have the fear of like, needing the validation from others to feel good enough. So our plan is, um, we take. So that's why we came up with the idea of making an Instagram account with the intention to help people from our school with Yuval, who fell into the trap of being a victim of social media. We created the account Walking mindset, um, to be reachable to people our age and who are in the same boat as us to be, um, we want to be a safe place for you and, um, with total acceptance to everything and everyone. Uh, we will promote our workshops also in the highlights you can see on our account. So, um. (...) Um, our contents will be, um, talking about people, people's experiences and their recoveries to help people who might not have the strength to, um, get help in person. Um, we will give people the space to open up and tell their stories, and we will also have special guests to post something and, and talk about their experiences, like social media activists who will talk about actual experiences and give professional advices there.

S2: Now, we already had, uh, the Instagram account and the content. The next step is reaching the community. Uh, by our target is the students in Royal Valley High School. Uh, we are going to creating the leaflets and put it on the wall. Uh, we also present about our information in, uh, homeroom classes. So the students can visibly see and know about our information. Then they can inform about us to another Asian student. After all the, uh, these implementation plan, especially the content in Instagram, uh, such as getting instruction from, uh, professionals or open up, uh, their story so they know that they are not alone and, um, getting their recoveries and many things. So this would make us reaching our goal, which is, uh, maintain usage of social media, which are people suffering. Thank you for the. (8) Thank.

(Black duck pitch, Pos. 1-6)

BLACK BEE (B1)

S1: Black be. [0:00:02.7]

S2: Black be. [0:00:06.0]

S3: Okay. Something to feel excluded in schools such as soaking our example. We the security. Selma. Annika, Bruna, Matsuko and me. We were trying to find a solution so people would feel excluded in school anymore. [0:00:22.1]

S4: So from our school experiences, schools don't really do integration among school classes itself. So we have come up with a plan to integrate more people and more teamwork inside classes. Um, our plan is to make activities that are non-school related during school time, like afternoons, maybe every six weeks or so. And it would include like card games, board games, playing football, sport activities, but something that's not really learning and to integrate people that might feel excluded. Um, yeah. So the whole thing should bring more people together and help their relationships inside the classes. Uh, our first step would be get in touch with schools and principals, managers, whatever the school has, maybe use their resources like already the games they have from maybe first two classes or so. And if it works in that class environment in this school, we would also teach the teachers, so to say, to make it themselves. [0:01:42.3]

S5: Yeah. So our idea should be implemented in schools because it's the it's a way to help to include people that might otherwise would have, um, a hard time of finding, um, like friends in class. It is simple, and it doesn't need much organization, um, to include this game day, um, in their schools, uh, routine. So while the game would be just a small one time investment, uh, you could really help people to, um, experience the school life without the stress of having to try to feel more included. [0:02:30.9]

S4: Thank you for your time. [0:02:31.9]

ORANGE DUCK (B1)

S1: Three. Two one. [0:00:04.2]

S2: So welcome to our project where the group Orange TAC and our project is called collective. We treat a problem number B1. So loneliness and I think a lot of us can relate to this problem. What do we all have in common? We're exchange students. Okay. And what did we all probably all have not. But most felt the first months maybe of your exchange. The first weeks. That's up to you. We had some moments of loneliness. There was one point for sure where you all felt like, oh, I'm so lonely, I don't have anyone. Well, I did, and when that happened, I always wished I had some place like the seminars where I could just go and have friends again. And that's why we love for kids that have maybe a bit of problems getting well integrated as cool. We thought of a summer camp that would happen a week before the start of the school year, and we thought it. Or at the time, age group. So it's not open to all ages just for the transitional ages, uh, between school grades. Okay. So for example, in Germany, that's between the fourth and the fifth class and between the ninth and the 10th, that would be giving this the students a chance to know each other beforehand and create stronger bonds and create new friendships, and B, in general develop social skills and in general, mental health retreats. [0:01:27.8]

S3: So yeah, by this we try to prevent loneliness, uh, before it even begins. And yeah, we're going to have multiple activities that should bring people together and create deeper bonds between them. First of them would be team sports. And to just have a fun and just have teamwork and some healthy competition. There also be cooking, which is great because it includes everyone. Everyone is apart even if they're not as good. And it's also rewarding because they get to eat it afterwards, right? Yeah. And it's not competitive. So like you're going to compete with. Yeah. And then we then we would split them into two groups. The younger ones would have some games and developed with the help of school counsellor, psychologist uh, which would target social skills. And the older group would be a bit too old for the games. So they would have conversation led by a school counsellor or psychologist. Yeah. Which would promote communication skills and create deeper bonds between them. [0:02:35.7]

S2: Like I said, this project is not about trading, it's way more about prevention. Want to cut the problem out even before it starts? That's why the kids are knowing each other beforehand. We're going to eradicate the problem before it even starts this we could. We have thought a lot about places. It could also be done just at school a week before because the school is still empty, you know guys. So or I use the center or another structure they only finances with me would be for, for example, for a structure which we're thinking we don't want to use, but just in case for food and ingredients for the cooking classes, for materials like the games. Because again. That the games are thought of are prepared by a school psychologist or counselor, and for material for the team. Sports maybe. Uh, other than that, we want to also say that again, um, the transitioning years of school is what we're mostly focusing on because we want the kids to not. Loneliness is a feeling that's always there. I mean, we got to feel a little bit some time before getting to the work, but we also want to have the kids learn a way to cope with it. So also learn how to be alone without suffering from. [0:03:58.4]

S3: And as the last thing is important to point out that it wouldn't be us, they wouldn't sleep there. It would be only during the day, which means the day parents can still work. And when they were in the work, the kids can just be there. And it also helps us with the costs. [0:04:15.3]

S4: It was kind of a thinking principle. Yeah. [0:04:18.2]

S2: So thank you for listening. [0:04:19.3]

PURPLE HORSE (A1)

S1: Number one for different courses. [0:00:11.8]

S2: Okay. Good morning. We are here to present Love Notes project aimed at addressing the negative impact of social media on the self-esteem and well-being of teenagers. In a world where social media platforms dominate our daily lives, it has become increasingly challenging for young people to navigate through the flood of idealized and false images that permeate online spaces. We all know the feeling. We scroll through our feeds and come across images of people with seemingly perfect bodies, flawless faces, and enviable lives. We begin to compare ourselves, our appearances, and our accomplishments to these unrealistic standards. We start questioning our self-worth and feeling the need to change ourselves to fit these distorted ideals. We begin to forget how to appreciate our unique qualities and the things we once loved about ourselves. Love notes aims to break down these false and idealistic images by fostering self-love, acceptance, and critical thinking among teenagers. Our project proposes the implementation of mandatory bi weekly workshops in schools. Students will be joined by young volunteers passionate about promoting a healthier relationship with social media. The workshops will combine individual work at home and group discussions in the classroom. Each student will be provided with a personal journal in which they will write daily love notes. These self love notes will consist of three parts. One thing they are proud of from that day, one thing they liked about themselves that day, and something they appreciated about their day. By engaging in this daily practice of self-affirmation, students will cultivate a habit of recognizing and celebrating their own unique qualities. During the workshop, students will gather in small groups to share their affirmations from their journals, creating a safe and supportive environment. They will then be asked to reflect on social media and why they post. This reflection will help bring awareness to their intentions behind their online presence, breaking down the taboo and fostering a deeper understanding of their own behaviors. By opening up this conversation, we aim to encourage individuals to become more conscious of their actions on social media and the impact they may have on others. Students will be encouraged to ask questions about social media topics they struggle with or find confusing. This interactive dialogue will allow for a deeper exploration of the challenges and complexities of the online world, further empowering teenagers to make informed decisions about their own digital experiences. In the final stage of our workshops, we will inform on misinformation in social media. Students will be shown how images can be edited and manipulated, and they will be presented with statistics that highlight the prevalence of edited photos and posed pictures. This segment aims to equip them with critical thinking skills necessary to discern between authentic content and fabricated representations. To ensure the success of love. Notes. We rely on, first of all, Yfu volunteers who will assist in facilitating these workshops. These volunteers will be selected from local Yfu communities where specifically passionate about our cause. They will receive a guideline to ensure they can effectively guide the students through the sessions to bring this project to life and make a lasting impact. We are seeking the financial support that will be used to allocate know to cover the cost of providing journals to students and inviting guest speakers who are experts in the field of social media. Background and its effects on mental health. Love notes strives to empower teenagers to look beyond the false and idealistic images presented on social media. By fostering self love, critical thinking and open dialogue, we hope to break the cycle of comparison and create a generation that is confident, resilient and aware of the manipulative nature of social media. Together, let's make a difference in the lives of young individuals and guide them towards a healthier relationship with the digital world. Thank you for your time and consideration. [0:04:01.3]**S3:** Next group is one. [0:04:02.9]

BLACK HORSE (C3)

S1: Pitch black horse. [0:00:03.6]

S2: Hey guys, have you ever been stress and anxiety in your life? Yes, actually, I've felt anxiety and stress coming out of school every single day. I have the feeling that I'm thinking and getting ahead in society. Or did you get all those J. Didn't you? How come? Yeah, actually we made a new project called Happy Mind. This is a project on the beginning of this of the starting line, though. On the rise. Exactly. These are going to be an app which won't only keep you away from your electronic devices, but in addition, uh, away from social media as well. Uh, while scrolling through thousands of videos, these videos are not only distracting your mind, according to the experts, but even deepen the anxiety. Happy minds helps us deal with these these problems by the medications, affirmations, self-care tasks and your excesses and a lot of things. There are a lot of things and go yoga okay does additionally reflecting and looking for help from experts? (..) Yeah, but how are you going to get there? The first thing we have to define a programmer, and additionally, this person has been found as the person or the project. And the person will be paid in a way of. Yeah. And our guess is going to be around €1,700. And. In the in the way of process. The last part of the process, we are going to finalize finalize the app in the company of therapist. And the therapist is going to be paid. Uh. (..) €600, and the last and the most important step to finish the project is to advertise the app to get the message across to the people which need to help and. Uh, is the most needed. And it was. My advertising is. Something about €100. [0:02:51.0]

S3: Thank you for your attention. [0:02:56.0]

S2: And this is our application image. And this is our logo. It's something you may. [0:03:04.0]

S4: Have asked how should it help? But imagine having an app that actually tells you to, hey, you have to relax because you're really, really tired. And hey, you became every time one task to care about yourself. For example, take a bubble bubble bath today or buy something sweet for you and something will will keep you excited and inspired for a whole school year. [0:03:35.8]

S2: And so we think that everyone feels stressed even in your exchange year, right? But it's normal and we are going to make this happy mind application to help you guys to deal with it in a better way. But attention, this application will will only be available to iPhones. So sorry for Android users in the others. (..) So. But I will use this, install this application and have a happy life and a happy mind. Thank you so much for listening. [0:04:19.4]

BLACK IGUANA (B1)

S1: Pitch black iguana. [0:00:03.0]

S2: Hi, I'm Sophie. This is Sophie, everybody. She's a high schooler at your local high school, and she's experiencing problems like loneliness and isolation. Yeah, and that's. [0:00:15.7]

S1: Why we had the question about loneliness in school and the like and presenting it. Our solution for you guys today, for that, we have an app called Body Language where you can find friends. And on that app we will have challenges where you, for example, wear a certain color of t shirt in your school to see other users and make new friends. [0:00:39.9]

S3: And we want the app to be safe and secure so that there's no weird people. So there's like people your age with similar interests. So it's like a safe place for everyone. [0:00:53.9]

S1: Um, and we're also going to have some volunteer counselors who are going to work with, uh, messaging with you that you can always text them and talk to about things that you are afraid of, talking to someone face to face or just without being afraid of being judged. And to our main point with this app, you can meet with new people that you thought that you maybe have no common interests and that are also in your local school. Um, and you can let them be your buddy. [0:01:32.6]

S4: Uh, yeah. So, uh, the back of the stuff, uh, the starting point would be to make a design, uh, of the app on paper. Uh, then give it to a app developer on a site like Upwork, where you can hire people for €10 per hour. Uh, then the next is developing the app itself and testing it for bugs and, uh, then we'll have to advertise it before publishing it. So it already has at least a baseline of, uh, users who will want to use it. Then we'll, uh, uh, approve the final version of this app. Uh. Uh, will. If we don't like anything, something, we will just be able to change it on the go. Uh, and then, uh. Yeah. And then we publish that the impact of our project should be a depression, and the loneliness rates are decreasing. Uh, the outcome is that people who don't have friends or people who are experiencing loneliness and isolation will find someone to talk to. And, uh, hopefully they'll find a body. And the output is, as they mentioned, is an app which will match with people with common interests. And yeah. [0:02:50.2]

S3: But of course, an app costs a lot and takes a lot of time. So in the beginning, to help Sophie in her school, exactly. In her school, we would start with a simple Google form that students can compile using the QR code. They can scan it, and you can access only with your school email so that no freaks around the school. Just the school. And just remember that if you're feeling like Sophie, everybody needs somebody. [0:03:19.4]

BLACK DUCK (A1)

S1: Route one black duck. [0:00:09.0]

S2: Yeah. So your time will officially start now. [0:00:15.5]

UU: So. Hello. Welcome to our. [0:00:18.7]

S2: Presentation. Uh, we are from Black Box Group, and our project is crack in mind. Uh, the problem that we choose is about, um, how might we empower the youth to cultivate, uh, a healthier relationship with social media? Let's get started. [0:00:36.9]

S3: Okay, so our problem is that too many students are badly influenced by social media due to a lack of how it actually works. So there will be times that you feel good and included in everything. And there will also be times that you feel like not being good enough that will lead to consequences, which can be, um, starting with building up an insecurity or. All the perfect body images you see on the internet can lead to mental health issues like having anxiety or something. To be scared to show yourself to others. Um, and it will lead you to a kind of obsession with social media, like having the urge to look like all of the perfect bodies that you see on the internet. After all, you can have the fear of like, needing the validation from others to feel good enough. So our plan is, um, we take. So that's why we came up with the idea of making an Instagram account with the intention to help people from our school with Yuval, who fell into the trap of being a victim of social media. We created the account Walking mindset, um, to be reachable to people our age and who are in the same boat as us to be, um, we want to be a safe place for you and, um, with total acceptance to everything and everyone. Uh, we will promote our workshops also in the highlights you can see on our account. So, um. (...) Um, our contents will be, um, talking about people, people's experiences and their recoveries to help people who might not have the strength to, um, get help in person. Um, we will give people the space to open up and tell their stories, and we will also have special guests to post something and, and talk about their experiences, like social media activists who will talk about actual experiences and give professional advices there. [0:03:00.4]

S2: Now, we already had, uh, the Instagram account and the content. The next step is reaching the community. Uh, by our target is the students in Royal Valley High School. Uh, we are going to creating the leaflets and put it on the wall. Uh, we also present about our information in, uh, homeroom classes. So the students can visibly see and know about our information. Then they can inform about us to another Asian student. After all the, uh, these implementation plan, especially the content in Instagram, uh, such as getting instruction from, uh, professionals or open up, uh, their story so they know that they are not alone and, um, getting their recoveries and many things. So this would make us reaching our goal, which is, uh, maintain usage of social media, which are people suffering. Thank you for the. (8) Thank. [0:04:16.8]

BLACK ELEPHANT (C3)

S1: Page one. Round one. Black elephant. [0:00:08.9]

S2: Is everyone up? Who needs to be in the front? Yes, yes. Perfect. Then let's get started. Your time starts now. [0:00:17.9]

S3: So we all have those days where we could really use a positive note in school, but it's so hard to find. Find it in your everyday school life. So we as a team had so many ideas and that we struggled a little bit to concentrate these many ideas in one project. So we came back to the basics and we as a group wanted to give every student in a school the opportunity to read a positive note and let it enlighten their day. So that's why we invented the Posie box, and it's a very good way to spread positivity. You put in one nice note and you take one. So it's a really good exchange. And it can really help you feel comfortable at your school to make your day, because sometimes you're just feeling down and you just want to read something nice. And it's a really good way just to make you feel better. And the puzzle box, um, is a better way than just the school toilets where it's written in the walls. Um, so. Yes. [0:01:31.2]

S2: Yeah. And it takes very, very little time. It's just come write a little notes limits. And if you want, you can receive an answer. So how would we get started? We cannot just go into schools and leave a box there. That's not going to help anyone. So we would work out a plan how to, uh, raise, um, uh, knowledge, uh, awareness about self love and through educational help to the school before putting up the boxes. So the teachers and the students and the school staff would really understand why it's so important. Um, we would so equip them with the knowledge, um, to understand a bit more. Um, so we'd set up many, not many, um, every school. Yeah. In the school, in different areas. Um, so many boxes. So you wouldn't have to walk to the other side of the school to leave a note. It would be very accessible. So hallways, entrance, the, um, the. [0:02:52.0]

S4: Classrooms. [0:02:52.8]

S2: The classrooms. Um, then we encourage the school to participate and remind them that it's really a very cool thing. Um, and to give, um, we would like also for the students to give feedback to, um, the teachers and the school staff by the class meetings that we all have in schools every other week, to just give feedback if there are any problems or any. Negative things about the box to for us to so perfect our idea. [0:03:37.8]

S4: Um. [0:03:39.6]

S3: Yes. So this is our puzzle box. And you leave a note and receive a note and it can make your day. And so to, to. [0:03:53.8]

S2: Finish off, uh, our whole exchange here for now, we would love for you to take notes. [0:04:02.2]

S5: If you want. Thank you very much. [0:04:13.5]

S6: It's just the first. [0:04:13.9]

BLACK FOX (B1)

S1: Everyone. Patron one black fox. [0:00:08.0]

S2: Then we shall begin with the black boxes. Time starts now. I have noticed. You have to sing. I have to sing. [0:00:16.8]

S3: One of you like the books? Yeah, I love me too, I love reading. What's your favorite? Uh, I don't know. [0:00:22.1]

S2: Like, um. What's the last book you read? [0:00:24.9]

S3: 1984. [0:00:26.1]

S2: Oh, my God, I wrote the book, too, I love. [0:00:28.2]

S3: Oh, really? [0:00:28.9]

S2: Yeah. It's like one of my favorites. [0:00:30.5]

S3: Your name? [0:00:31.3]

S2: And my name is Olivia. What's your name? [0:00:32.7]

S3: Santino. We have to do something together. [0:00:34.3]

S2: Yeah, that's really nice. (..) Oh, my God, I'm so sorry. Oh, my God, I'm so sorry. That's fine. Um, yeah, I, I don't know what I'm doing here. [0:00:46.9]

S3: We are talking about books, you know? [0:00:48.5]

S2: Yeah, like we have the same sticker. So I went up to stickers. I have one. Yeah, I have actually one. Like it's sports. I don't know if you like sports. Yes, I like sports. So here you go. You can have it, put it on your phone so people like, read it like right now you can speak and find friends. Good. Thank you. Um, okay. I hope you liked our little eggs. And you have now an idea of what we're going to present to you. Just start loneliness. Everybody goes through some kind of loneliness. Uh, whether it's in school or doing the exchange, you're like, we did experience some loneliness during our exchange here. And that's why we want to help those people. Because we know what it feels like. [0:01:32.2]

S3: Yeah. Now, I wanted you guys imagine the next situation. You are in the middle school, and maybe you are not shy people or something like that, but also you have problems to make friends or to find people with the same hobbies as you. [0:01:46.6]

S2: And that's why we created a social sticks, which which stands for socializing and stickers. So our idea is to create an Instagram account which educates people about loneliness and makes people aware of what it is to feel like and what people can do to overcome it. And that's our second idea. The stickers, like you already could see we want to use the stickers as a conversation surface, and that people could find people with same interests. So let me explain what the sticker actually work. So here you can see that our sticker is in the shape of a circle and on each stick, and made with eco friendly paper on a sticker, you. [0:02:30.2]

S4: Will see there's a type of hobby. For example, we have reading and we have sport, and we also have gaming and so on. So um, so basically from the beginning, the example, you will see it just like a, um, we, we have a, we have a chance to recognize the people who have the same hobbies as you. And I get a chance to start a conversation and maybe get a chance to have a new friend. And then we're going to hold some clubs, and then we will put them on our Instagram account, and you will check the time and location on our account. [0:03:08.1]

S2: And yeah, it's the the impact we want to make is really the people take the courage, go to the other people like we did and speak and overcome their anxieties, fears and make new friends. [0:03:22.4]

S4: So what we're going to do is more and more people start to know our account and start to use our sticker. We're going to extend it to more communities and more communities. We know that. And then we're going to host more clubs. And, um, and so don't be shy to ask us about your suggestion or any problem you are facing. And then we are always here waiting for you. [0:03:48.1]

S3: So in conclusion, that was our ideas and our arguments to find a solution to decrease the loneliness around our community and the different schools. So thank you very much. Yeah. (6) Thank you. [0:04:06.8]

BLUE DUCK (A1)

S1: Blue Duck's pitch one. [0:00:03.4]

S2: Have you ever felt tricked or cheated? It might not be a pleasant experience, but it sure is memorable. [0:00:10.9]

S3: Hi everyone, I'm David, this is Flora and we would like to present to you a solution to a problem called social media. And how might we empower the youth to cultivate a healthier relationship with the social media? We want to implement our project on a kids aged between 12 and 15 years old, to help them recognize the fake side of the internet and develop a healthier perspective on social media. [0:00:40.5]

S2: Our plan is to visit schools as a fake company and do an experiment. Our fake company would do serious seminars about self-acceptance and body positivity in connection with social media. These sessions would be for the students and the parents can also participate. We would say that this usually costs €50, but this time and this only special time is free. We have only one condition that we would like to film a commercial while it's ongoing. We would have. We would have everyone's written consent to being filmed beforehand, and then we would be doing this very serious seminars for two hours and will also stay for the breaks. Then when it's all done and we have enough footage, we'll pack our equipment together and leave. Now comes our editor, or our editor will make our commercial, which will which will showcase a very different experience. Our seminar was serious, but the video will be fun and upbeat with lots of laughing music. [0:01:39.3]

S3: We're also going to have fake interviews with the kids where we're going to ask some questions. That's going to have a happy answer, and we're going to completely change the context of the video and make it seem like there's a really big contrast and difference between the video and their real experience. Later that day, we're going to show them the video that's completely different from their expectations, and we hope that they're going to feel like surprised, used, tricked, maybe angry at us. And that's the point. When we're going to reveal ourselves, we're going to say our point that we will that we will prove that's the point, that everything on social media hasn't be right. And they have to check everything twice for the contrast. We're going to show our real video as well, which is not edited. And there they have to see the difference and contrast and they should learn a lesson from it. [0:02:40.4]

S2: The budget for this project could be different depending on which country it is facing. We thought that us or other volunteers could do the seminar part, so we only need an editor and a cameraman in Germany for these two last tasks. Uh, with the wages and our time management, we think we could do this project in two different German schools. [0:03:02.0]

S3: So, yeah, we believe our project could be really memorable, and it could teach the kids a really important lesson for their lives that would last really long. So I hope you're on our board as well. And thank you for listening. [0:03:24.1]

S1: Blue Duck's Pitch one. [0:03:25.7]

BLUE ELEPHANT (B1)

S1: Blue elephant speech. [0:00:01.4]

S2: My mother makes a lot of very great projects, and there's this one project that always stood out to me. It's called Spirit of Christmas. What is Spirit of Christmas? You might be wondering. Spirit of Christmas is a project where you go in seniors homes with gifts that were donated like gifts, foods, necessities, needs and wants from the community. Then we go visit seniors that haven't had visitors in five or so years. They're lonely. They have no one, and we go there to make their Christmas fun. And you're wondering what does like what is a senior in need? So like I said, is seniors who haven't seen anyone, their families now have contact with them. It's just seniors who are very lonely. And seeing this, I realized that seniors and teenagers are quite alike. We're very lonely. We don't have a lot of friends. We don't have a lot of ideas. We don't have a lot of things to do when on our own time. So with this background knowledge, I brought it to the group and we created the idea called scenes with teens. [0:00:59.0]

S3: So continuing from that, we can see that there was a study made that says that loneliness has the same health effects than taking 15 cigarettes a day. So when you think of teenagers, we think of lively and we're constantly communicating with each other. Um, but that's not always the case yet. There's like, for example, here. Yes, we're all here to connect and make friends, but there's always some people that will find it a little harder to fit in. So, for example, um, this is really hard for some people that affects other mental health. Um, and the feeling of isolation really, um, affects the mental health from all of these people. So that's why we created this graph to show, um, the how loneliness affects, um, different ages. So here you can see that really like all the ages are really, um, affected by loneliness, especially the teenagers and the group that we found out that we don't talk about enough is the elderly people. So we decided to make scenes with teens. Um, it's a project that we'll be doing in a local school. We'll be taking a class, um, who will be taking a survey, the teacher will be making a survey, and everyone will fill out the survey based on their, like, personalities, and they will be matched up with someone with their same personality, basically as a way to make a new friend. And then they would be able to go to a seniors house almost every other week to meet a senior or an elderly person and to connect with them. So two students will each be paired with one senior, and we'll get participations in the nursing home by hanging up flyers for the seniors, where we're going to ask for people to talk about their experiences with loneliness. So then they would meet up every second week. But we would also use the money provided for fun trips to spend more time together. That would be monthly, and those would be trips like going together to a museum or watch sports events. And then, um, at the end, the participants, like the students, will also be asked to do a presentation about the seniors they spoke to and share the experiences they got from them. So, um, through all project, teenagers will get the opportunity to engage with new people and hopefully close relationships and bonds will be formed. But not only friendships between teenagers and seniors, but also between two teenagers that are very alike with the same interests. And we will reach that through our anonymous surveys in the classes. Um, hopefully the teenagers. Um. (..) Hopefully the teenagers, um, would develop new skills by understanding the feeling of elderly people who might feel isolated or lonely. With our project, we will bring together two groups of people. People who are very different. They will get together, get to know one another, and can learn from each other. Thank you. [0:04:19.6]

S1: Blue elephants, page one. [0:04:21.4]

BLUE FOX (A3)

S1: Blue Fox pitch one. [0:00:03.4]

S2: Have you ever looked into a mirror and felt like you weren't enough? Don't worry, you're not alone. As studies have shown, 85% of people struggle with self love issues at some point in their lives. Unfortunately, this issue of self love is present, especially amongst us, the young people. That's why we from Inner Bliss started this project that contributes to a sense of self exploration in young people. [0:00:42.2]

S3: I want to introduce you to Inner Bliss, a transformative website designed to help you embrace self love and overcome self-judgment in a world that often fuels self-doubt. Interval serves as a virtual space where you can embark on a journey of self-discovery, self-acceptance, and ultimately, self-love. The website is easy to reach, flexible for everyone, and fun to use. Our diverse range of creative resources including videos, practices, activities guide users on a journey of self-discovery and acceptance. Our engaging videos inspire users to build self esteem, nurture a positive mindset, and practice self care through active participation in practical exercises like journaling and guided meditation. Individuals explore their thoughts, emotions and personal growth. We will also be active on social media, creating accounts to reach more people through their For You page and fees. The impact of intervals extends beyond individual wellbeing. When individuals learn to love and accept themselves, they radiate positivity and become a source of inspiration for others. As Enablis users experience personal growth and empowerment, they nature, they contribute to building a more compassionate and supportive society. Now let's shift our focus on sustainability of enactus. Sustainability is a core principle that guides every aspect of our website. Firstly, Enablis is designed to be easily accessible for anyone with an internet connection. We prioritize user experience and ensure that the website's intuitive, user friendly and compatible with various devices. [0:02:35.0]

S2: One of our next steps is for a project to get popular enough to reach out to influencers and hopefully collab with them. We would like to show people that influencers whose life seems perfect to us are real people with the same problems as well. To do that, we would like the influencers to share struggle stories from their personal lives and explain how they got over their problems. Their popularity could really help us shine a light on this important problem of self-acceptance. Our priority is not to win. We've already won by standing here in front of you and presenting this very hard and personal problem. We're very grateful for this opportunity. Unfortunately, this project is very doable with a without a big funding, but a larger budget would mean reaching a wider audience and raising more awareness, which is our priority. If there's one thing we want you to remember from our presentation, is to always be kind to yourself and focus more on your personal needs. We hope that we inspired you and that we can keep inspiring you in the future by making Établis a reality. Thank you for listening. [0:03:38.8]

UU: And the Blue Fox phase one. [0:03:41.6]

BLUE GOAT (A2)

S1: Growing concern amongst students regarding appearance based on goals, and this decreases the concentration we have on our overall health from physical to mental health. This is where our app comes in. This is angles. [0:00:22.6]

S2: To tell you about some of the core features we have. For example, the info page where you can educate yourself, build on the topics with articles, and test your knowledge out there in a fun quiz to keep the everything fun and nice. Um, also for physical health, we have recipes, we have workout videos, and more. One of the most important features is the calendar. It's a calendar where you can see events and actions happening in your area. To go out and build a strong sense of community. [0:01:03.0]

S1: But that's not it. We have a special thing that we thought about, and this is from my personal experience, and it's called the casual magic, because in our lives we all have casual magic. It's you. You are the casual magic. It's where you gain all your confidence. You have affirmations every day where you say, I am enough, I am beautiful. And that's when you believe that. That's when you think I'm enough. We also have a habit tracker where you see how your life changes. This is where we see if our app did help you. We have also a hotline. This is for more emergencies for each country. We're going to add the hotline you need when you need an emergency and you can easily find it. It's going to be a space where you find the help you need. And lastly we have the journal. This is a space we created for you to have a safe place to say all the things you want to say, and they will be automatically saved and automatically delivered to you six hours after. So you can see how you felt throughout the day. [0:02:24.1]

S2: But the question is why now? We thought nowadays, the easiest way to target people in our group, in our age group is finally, we think it is kind of the problem that people are already so much on social media and on their screen. So we wanted an app where people don't spend the most time of the day. So we implanted the calendar to people, spend time outside and go out and join the community to realize this app. Without about the funding, the €500 funding, we would invest in getting some technical expertise to help us build and create this app. (..) To show, to see, keep track of our impact of them, we thought about a controlled study where we, a certain group of people use them, a certain group of people don't use them, and after a while they're going to answer a few questions to see if the app has a positive impact, negative impact, or don't. [0:03:34.9]

UU: Have an impact. Thank you very much. [0:03:43.8]

S1: Blue Goats, which won. [0:03:45.7]

PURPLE ALBATROSS (B3)

S1: Purple albatross. [0:00:03.7]

S2: Where? Purple albatross. My name is Owen. [0:00:06.0]

S3: Hi, my name is Nya and I am Madeleine. Are you or do you know someone who is neurodivergent? [0:00:13.0]

S2: What does it mean? [0:00:14.0]

S3: Neuro being neurodivergent means that one's brain processes, processes, information and develops differently from a typical brain. Actually, my own brother, he has autism and ADHD. Oh, my best friend has ADHD and epilepsy and also KD. Do you know anybody else who are neurodivergent? [0:00:33.1]

S2: Well, I would say I know myself pretty well, I am neurodivergent, I have narcolepsy with cataplexy. [0:00:38.1]

S3: Wait, is that the disorder where you like sleep a lot? [0:00:41.4]

S2: Yeah. That's it, but it's a bit more complicated than that. A lot of people don't really know what it is. They often think I'm just lazy or get confused when I randomly fall asleep in seconds. [0:00:51.2]

S3: Okay, well, it seems like we all know somebody who is neurodivergent. It may be my brother, your best friend, the friend of a friend or a friend, a neighbor or even yourself. Um, so we are all affected by this? Yeah. It is such a common thing. Yet at least I sometimes find myself in a situation where I don't know how to help these people. I don't know what to do in these situations. And it all starts from educating people. We need to educate people, and because the more we know, the better we understand and the better we understand, the more we know what we can do in these kinds of situations. We want to help these people make them feel better and and as comfortable as possible. And our focus is in the school setting. [0:01:38.2]

S2: It is crucial to create an environment that supports their learning styles. They might feel really overwhelmed, anxious, or have a hard time focusing. So we want others, teachers and students to understand the situations as good as possible. [0:01:53.3]

S3: Yes. And how are we planning to educate these people? [0:01:57.2]

S2: Well, our plan is to do guest lectures, offer the possibility to call professionals to schools and inform and educate people on a specific topic, and also invite neurodivergent people themselves to share their experience and lifestyle. [0:02:10.4]

S3: But to reach a larger circle of people, we want to create social media platforms to be able to educate people. Yes, and these posts would be from different kinds. So we would include statistics. We would include people who can share their personal story and also, um, explain the accurate definitions of the different terms that are around neurodiversity because a lot of people don't know them, and also step by step help guides. [0:02:41.7]

S2: And of course we promote the guest lecturers. [0:02:44.0]

S3: One other idea to promote our work is to use QR codes, just plain stickers. We'll just slap them on the wall and the no name, no nothing. And just especially in school, we put them as it's yeah, it's effective. [0:02:57.9]

S2: Yeah. Because we think that this way we can wake up the curiosity in people. [0:03:02.7]

S3: Yeah. Because if I personally see a random QR code on a street, I will go and scan it. I mean, I have done it, it works. It just gets me every single time. And where does this QR code take you? Directs people to our web page, and from there, people can go further onto our social media pages and they can, um, invite professionals or neurodiverse peoples themselves to their communicate community to help them educate their classmates or their their community. Yes, and to introduce the rest. The other brains behind our project. I'm Ellie. [0:03:38.8]

S4: I'm Megan, and I'm Bethany, and sorry, I'm Darren. [0:03:44.0]

S3: I'm Zuzana. [0:03:45.3]

S4: And this is our logo. And the lying eight or the infinity sign is also known as the sign for the awareness about autism. And we included the wings because our project is called purple albatross. And an albatross is a bird. So we included the wings to kind of grab this information again. And we hope it's really appealing. [0:04:08.6]

S5: And this is an example of posters we would make. And they would also include a our contacts and our social media and posts. [0:04:18.6]

S3: Yes. Yeah. There was something yeah we're a purple albatross and we thank you. [0:04:23.9]

PURPLE BEE (B3)

S1: This is from the Purple Beast. Okay. Hi, everyone. We're the Purple Beasts, and we're here to present you today our pitching on value of diversity in school systems for this, uh, learning disability. People, for example, people with dyslexia, ADHD, autism or dyscalculia. And we chose this project because there's some people in our group that are suffering from one of these or few, and we don't think that we're getting enough help for us. And then some of us are just concerned that there's not enough, uh, like, uh, stuff that are made for us to feel included in the school system as well. So our project is going to. [0:00:47.1]

S2: Be, um, a, uh, workshop where experts come in and explain the disabilities to the students. And afterwards, the students actually get a chance to interact with the problem by solving little exercises with, like, ADHD, where they have to do a reading comprehension while there's loud music playing or a video, um, playing in the background or with dyslexia, they have to try to read a text on a laptop where the letters are moving and the sentences are keeping, like keep mixing up. And with autism. We thought about making a video of the POV of an autistic person and showing it as a presentation. Our aim of this project is that the disabilities are getting, um, shown closely to the students to raise awareness and understanding and to make people, uh, that suffer from this disabilities feel more included. [0:01:56.2]

S1: Okay, so what do we need to make a project a reality? First of all, we need like professional ads, for example, uh, psychologists to supervise our workshop, but also to help us to set all the activities. Then we need respectful students to, um, who are ready to discover all the activities and a new point of view of what the life could be. And at the end, we need teachers to, um, supervise all the students and, um, just to make sure that all work. So, as Ada said said, for the workshop, we have planned a lot of activities and for example, for the activity of, uh, ways the dyslexia, um, we'll need a computer to read all the texts. [0:02:48.9]

S3: For example, for our ADHD workshops, we need experienced teachers for us to, um, that worked with people who struggled from ADHD with, uh, people, I mean, kids as energetic as ADHD kids. Patience is key. We would also need some isolated and separated rooms for us to make, uh, open some music and for there to be no distractions. [0:03:15.2]

S1: For the autism. We want to, like, present the video. So we'll need a Beamer and an Ola to show the video. [0:03:22.6]

S3: For Dyscalculia Workshop. So we would actually use the same concept as this dyslexia. But instead of focusing on letters we will focus on numbers. [0:03:33.0]

S1: Okay, now for the budget. We are hoping to find volunteers because our project is based on the education and we want to have the support of the schools, but also of the government. So we hope to find a volunteers. But if not, we'll see like the budget in the moment. [0:03:51.6]

S3: And lastly, I want to talk about the obstacles that we could face on our journey. So it's obvious that working with children is always hard and we need to motivate them with our energy, just as Yafeu does to us with their seminars and, uh, these kinds of energizers. Another problem might be schools that don't have enough place and space for us to create some workshops, and they might be also kind of ignorant for these kinds of learning disabilities, and they might just not want to come with us in our journey. Um, to sum things up, we want to talk about talk with schools and bring some

experts, uh, to spread awareness about learning disabilities and help the students who are struggling from learning disabilities. Thank you for listening. [0:04:37.2]

PURPLE CAT (C1)

S1: Cats. [0:00:02.8]

S2: Hello and good morning everyone. Today we're going to talk about stress, more specifically stress in schools because very often students feel extremely pressured by their workload, the pressure of the teachers or their families put on them, or even the pressure they put on themselves when it comes, for example, to their grades. Normally, schools are not aware of this insane problem, and we want to change that with our project. The name of our project, the most important thing is lose the stress and the target group are 13 to 18 year olds. So the age group we normally find in high school, the participants or the people who participated in creating this project are Laszlo, Ana, Jeevan, Anita, Bio, Sarah, Elio and me Valentina. And the question is or our goals are to spread awareness and empathy in schools and to make them a more comfortable place for the students, for the teachers, for everyone. Now the question is how do we want to do that? And Sarah is going to answer this question. We plan to do that by creating a workshop. And the first part of the workshop is going to take three, 2 to 3 days. And we're going to work with professionals to teach the students about the effects of stress they can have later in life, and also learn about coping mechanisms if they have, like extreme stress and learn their preferred study methods, how to manage time and just reduce the stress they already have. Um, we also want to implement a little project that's going to take half a day, maybe a day, where the students can design their perfect school in, uh, in groups of eight people, maybe, maybe less six people, and just give feedback what could be done in their school to make it better and less stressful. The next day would be an evening for the teachers, where the teachers are going to get the feedback the students gave in this project, the make a better school. Um, and we will see with them what they can improve in their school, what is possible to do with the funding, the capabilities they have, because most schools lack teachers. So we're not it's not possible to, um, implement all the ideas, but we'll try to do as many as possible. We will also help the teachers educate them about stress and how they can manage their assignments, their project better, and spread them out of the whole year. Instead of having 1 or 2 exam weeks for all the all the exams are crammed in, the next day would be for the parents, because parents often dismiss the stress we as students have because they are busy with work. So we want to also teach the parents about the stress. Even, uh, their kids can have any young age. For that, we would try to collect as many stories about extreme stress from the students as possible and read them to the parents so they know what kind of stress their children have in that age group. So in conclusion, the in our opinion, best way to, like I said, spread these awareness and empathy and to make the schools really a more comfortable place is to ask the students how to change the schools, what they want to change, what they think are the problems, and what they may give as tips for other people who are struggling. Thank you for listening and have a good day. [0:03:41.0]

PURPLE DUCK (B3)

S1: This is the purple ducks. [0:00:03.2]

S2: So hello everyone that have gathered here for this special occasion. My name is Leon Tamal and I will present you the Purple Duck organization. Before starting, I would like you to close your eyes and imagine the following situation. You are in a classroom about to write an exam and you get your paper. You're trying to read the first question, but you're not capable. You're looking over at your classmates and they are all already answering the question. But you are visually impaired and the text is too small for you to read, so you are not able to show your knowledge on a test just because the exercise didn't take into consideration your disadvantage, wouldn't that make you feel excluded? You can open your eyes. These are the problems that neurodivergent individuals struggle with at school. This is why we founded the Purple Duck organization. So what is this organization organization about, you may ask? Our goal is to spread awareness about neurodivergent disabilities in our education system. We want to include the students with cognitive differences in the class and the class community, as well as preparing them for future job prospects. There are many different ways we are going to spread this awareness. Firstly, we would create a community of volunteers that have had the necessary formation on the subject sub subject. With the help of these volunteers, we ensure the general comprehension of the everyday struggles of people with neurodivergent difficulties to the general public. Secondly, by effectively taking advantage of the major influence of various social media platforms, raising awareness about the subject will be easily manageable. Content creation and the flow of relevant information is the key to destigmatize, clarify and answer questions about neurodivergent disabilities. So how would that look like in practice? You might ask? Our motivated volunteers will create a suitable environment by pitching and advertising in school, an environment that promotes active listening, inclusivity and creative approach. The usage of interactive simulators, for example, VR simulators, videos that are simple to follow, displaying practical information on posters and leaflets are all proven methods of raising awareness. A major part of our project will also include the organization of public assemblies that will take place under our supervision, and will be completely moderated by professionals and people that can share their own experiences. By implementing these methodologies methodologies, we assure a better environment and an economical and an effective approach to dealing with soothing the struggles of people with neurodivergent difficulties and disabilities. [0:03:02.1]

S3: So why should you choose us? When we came up with our idea, we wanted to make a lasting impact. It is estimated that as much as 15 to 20% of the population are struggling because of neurodivergent disabilities. Our vision is to help these people and this community, and with your support, we can make an even bigger difference. Thank you. [0:03:27.0]
