# Supplementary material for: Co-Creation of Mental Health Intervention for Adolescents: A Social Hackathon Approach
Source: Healthcare (Basel). 2026 May 12;14(10):1315. doi: 10.3390/healthcare14101315 (PMC13205654; doi:10.3390/healthcare14101315)
Supplement: Supplementary file 1 [file healthcare-14-01315-s001.zip › 6. alluvials diagram interactive HTML.html]

Alluvials


Round 1

Round 2+

Round 3+

Round 4
